# Visible Light Spectroscopy of Liquid Solutes from Femto- to Attoliter Volumes Inside a Single Nanofluidic Channel

**DOI:** 10.1021/acsnano.4c15878

**Published:** 2025-01-07

**Authors:** Björn Altenburger, Joachim Fritzsche, Christoph Langhammer

**Affiliations:** Department of Physics, Chalmers University of Technology, SE-412 96 Gothenburg, Sweden

**Keywords:** nanofluidics, nanofluidic scattering spectroscopy, attoliter volumes, Kramers–Kronig relation, concentration measurement, reference scheme

## Abstract

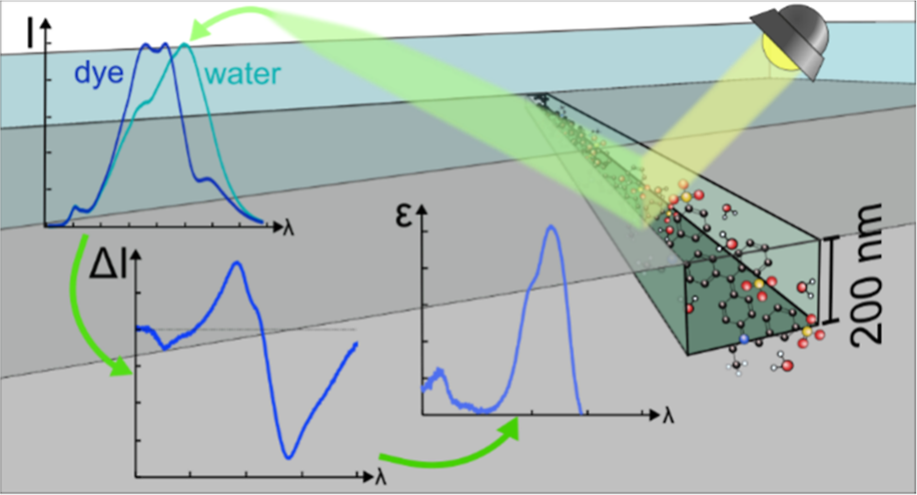

UV–vis spectroscopy is a workhorse in analytical chemistry that finds application in life science, organic synthesis, and energy technologies like photocatalysis. In its traditional implementation with cuvettes, it requires sample volumes in the milliliter range. Here, we show how nanofluidic scattering spectroscopy (NSS), which measures visible light scattered from a single nanochannel in a spectrally resolved way, can reduce this sample volume to the attoliter range for solute concentrations in the mM regime, which corresponds to as few as 10^5^ probed molecules. The connection of the nanochannel to a microfluidic in-and-outlet system enables such measurements in continuous flow conditions, and the integrated online optical reference system ensures their long-term stability. On the examples of the nonabsorbing solutes NaCl and H_2_O_2_ and the dyes Brilliant Blue, Allura Red, and Fluorescein, we demonstrate that spectral fingerprints can be obtained with good accuracy and that solute concentrations inside the nanochannel can be determined based on NSS-spectra. Furthermore, by applying a reverse Kramers–Kronig transformation to NSS-spectra, we show that the molar extinction coefficient of the dye solutes can be extracted in good agreement with the literature values. These results thus advertise NSS as a versatile tool for the spectroscopic analysis of solutes in situations where nanoscopic sample volumes, as well as continuous flow measurements are critical, e.g., in single particle catalysis or nanoscale flow cytometry.

## Introduction

Spectroscopy based on electromagnetic radiation is one of the most fundamental experimental principles in modern science and has enabled an uncountable number of advances in research since its formal description^1^ by Isaac Newton in 1704. For example, it has offered invaluable insights into the nature of light and matter due to a plethora of interactions between different mechanical or electromagnetic frequencies and the very basic structures of, e.g., solid crystals, molecules, atoms, and electrons. Investigating the frequencies of these interactions individually, i.e., in a spectrally resolved manner, yields information about structure, composition, electronic transitions, and chemical reactions that occur inside or on the surfaces of the sample the radiation interacted with or originated from. Because of this wide applicability and the wide range of information that can be obtained, spectroscopy based on electromagnetic radiation has become a workhorse in the experimental method toolbox of the natural sciences and has since its introduction diversified into a plethora of associated methods and techniques.

To structure these spectroscopic methods, we can organize them according to the size and volume of the investigated sample. To this end, the standard versions of spectroscopic tools typically enable investigations of macroscopic samples only, that is, samples that are contained in relatively large containers, such as cuvettes or gas chambers, or samples such as crystals and powders whose volumes are large compared to the actual molecules or particles of interest. These techniques can then be ordered by increasing frequency (or decreasing wavelength) of the electromagnetic radiation exploited to probe the sample, that is, for instance, microwaves,^2^ infrared radiation^3−6^ (IR), ultraviolet–visible (UV–vis) light, X-rays,^7,8^ and electrons.^9,10^

While all are very powerful in their specific regime, methods employing UV–vis light have earned a special place in this list since, e.g., many natural processes require the interaction of sunlight with (living) matter, which means that many biologically important molecules absorb UV–vis light.^11−14^ Furthermore, it is also the same wavelength range that is used for modern optical communication^15,16^ and as a source for sustainable energy.^17−21^ The main methods used in this spectral domain are absorption^22^ and emission spectroscopy,^21,23−25^ surface plasmon resonance (SPR),^26^ and localized surface plasmon resonance^27^ spectroscopy, as well as Raman spectroscopy.^28^ They, e.g., can reveal the electronic structure of molecules and transitions between different electronic states or electronic excitations and transitions in solids, such as metals and semiconductors, as well as small refractive index (RI) changes induced, e.g., by the specific binding of molecules onto plasmonic surfaces.

Considering the typical dimensions of the entities that interact with the radiation at hand, efforts to reduce the required sample size or volume to the micro- or even nanoscale have been a strong driving force in the development of new spectrometric methods. As key reasons, we identify that reducing the required amount of sample material is desirable both from a cost perspective when expensive/scarce raw materials are used and since the synthesis of large volumes of new materials or molecules often is very difficult and time-consuming and hence hampers the throughput in, e.g., screening processes. Furthermore, spectroscopic methods that provide micro- or nanoscopic spatial resolution (enabled by tiny sample volumes being enough to generate a measurable signal) make it possible to investigate nanomaterials, particles, surfaces, and molecules in greater detail, in a manner that can differentiate between locations and species, and ideally also beyond ensemble averaging. These prospects have driven the development of nanoscale spectroscopy methods,^29^ such as IR atomic force (AFM) spectroscopy,^30,31^ IR scanning tunneling microscopy spectroscopy,^32,33^ tip-enhanced Raman-spectroscopy,^34^ scanning near-field optical microscopy^35^ (SNOM), electron energy loss spectroscopy, cathodoluminescence methods,^35^ coherent phonon spectroscopy,^36^ single particle plasmonic spectral sensing,^37^ as well as many approaches involving fluorescence.^38,39^ It is evident from this list that many of these techniques employ either tip- or plasmonic nanoparticle/surface-based signal enhancement concepts often in conjunction with scanning microscopy and/or utilize the IR or ultrashort wavelength regime (X-ray, electrons) of the electromagnetic spectrum. Especially noteworthy for our discussion here are concepts that integrate surface-enhanced Raman spectroscopy with nanofluidics, as done with colloidal nanoparticles^40^ or nanoslits,^41^ enabling the detection of protein folding states and single nucleobases, respectively. Furthermore, the use of nanoscale hotspots in conjunction with surface-enhanced infrared absorption (SEIRAS) made it possible to quantify molecules in a nanofluidic system.^42^ While these results clearly demonstrate the possibilities of fluorescence and surface-enhanced Raman and IR approaches, we identify a distinct lack of nanoscale spectroscopy methods that are label-free, i.e., do not require fluorescence, and that do not require signal enhancement by plasmonic tips, surfaces, or nanoparticles. An interesting approach that relies only on the intrinsic properties of molecules to decrease the detection limit while omitting surface enhancements is given by photothermal optical diffraction^43^ and photothermal optical phase shift,^44^ both relying on the temporary variance in RI caused by the thermal de-excitation of molecules. However, these techniques do not provide spectral analysis in the vis spectral range, making it clear that a label and plasmonic enhancement-free nanofluidic characterization method for this wavelength range is missing. This means that to our knowledge, there is currently no method for nanofluidic applications that reproduces the performance of established macroscopic UV–vis spectroscopy.

To fill this gap, we present nanofluidic scattering spectroscopy (NSS) that enables label-free spectroscopy in the visible light (vis) regime inside individual nanofluidic channels. These channels offer tiny sample volumes, in our study here ranging from 2 fL to 60 attoliter (aL), depending on chosen nanochannel dimensions and settings of the CCD camera. This concept is a further development of Nanofluidic Scattering Microscopy we recently have introduced.^45,46^ As the key features beyond the state of the art, NSS is able not only to deliver the full wavelength-dependent scattering and absorption spectrum and thus the wavelength-dependent molar extinction coefficient of a solution contained inside a single nanofluidic channel but also to reveal its wavelength-dependent RI and the absolute concentration of solute. This, we illustrate on five examples of transparent, colored, and fluorescent molecular solutions in the millimolar to molar concentration regime. Furthermore, in contrast to similar techniques on open surfaces,^47^ since a nanofluidic channel connected to a microfluidic system is used as sample vessel, continuous convective flow is enabled and thus facilitates both high experimental throughput and the possibility to monitor changes in the probed solution continuously.

## Results and Discussion

**Figure 1:**
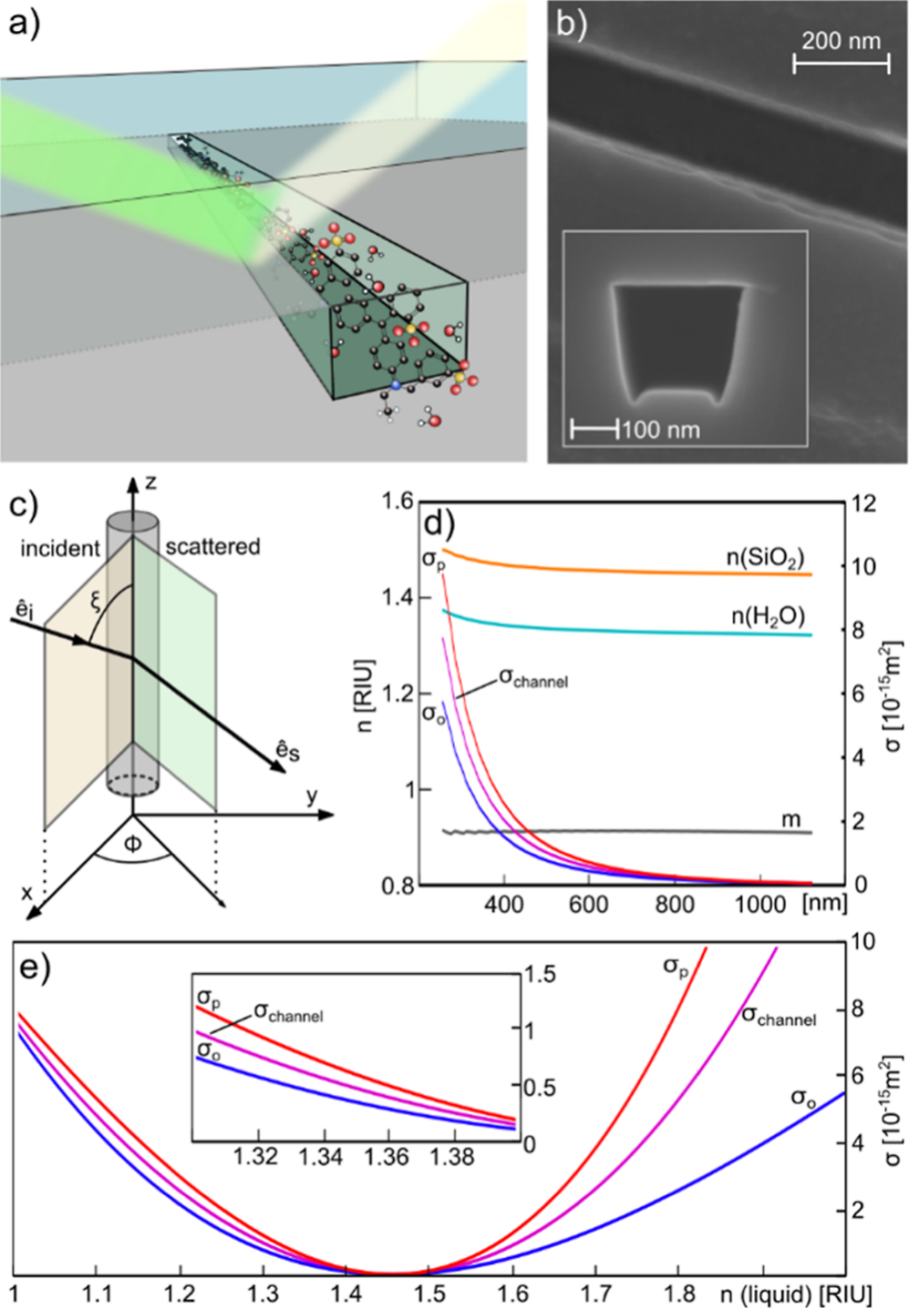
Principle of NSS. (a) Artistic rendering of a nanochannel filled with dye molecules. An incident light beam is partially absorbed by the dye molecules, partially scattered on the channel, and thereby changes its spectral composition (see Figure S1 for a complete sketch of the experimental setup). (b) SEM image of a 200 nm × 200 nm nanochannel. The inset shows a cross section of a nanochannel, being nearly rectangular. The small features are a consequence of the etching process. (c) Sketch of the analytical theoretical model used, with the nanochannel approximated as cylinder (gray) and the planes of the incident (orange) and scattered light (green). The Poynting vector of the light is shown in black. (d) Incident light wavelength, λ, dependence of the scattering cross section, σ, of a water-filled channel shown for a polarization parallel to the channel (σ_p_), orthogonal to it (σ_o_) and for unpolarized light (σ_channel_). Data plotted together with the wavelength dependence of the RIs, *n*, of water^56^ and silicon oxide^48^ and their ratio, *m*. (e) Calculated scattering cross section of a 200 nm by 200 nm channel embedded in silicon oxide (*n* = 1.459) as a function of the RI of a medium inside the channel, again shown for three different polarizations of the incident light at 600 nm (see Figure S2 for all visible wavelengths). The inset shows a zoom in of the RI region relevant for aqueous solutions.

Scattering of electromagnetic radiation from small objects or molecules is a phenomenon often encountered in nature. It is the reason why the sky is blue and also why tiny scratches in a window are visible. Mechanistically, those scratches scatter light because they are objects with a different RI than the surrounding glass (*n* = 1.459^48^) since they are filled with air (*n* = 1) and because they are smaller (Rayleigh scattering) or comparable (Mie-scattering) to the wavelength of the irradiated light. In analogy to scratches in glass, in this work, we make use of the light scattered from nanofluidic channels etched into a silicon dioxide (SiO_2_) surface and hermetically sealed with a glass lid (Figure 1a). The choice of SiO_2_ as substrate is well established within nanofluidics,^45,46,49^ as it offers great chemical resistance, and various fluidic designs can be realized using fabrication methods established for silicon-based microelectronics.^50^ Specifically, the nanochannels we use here constitute an elongated rectangular cavity with 200 nm width and depth, and 62 μm length, nanofabricated into the 250 nm thick thermal oxide layer of a silicon wafer (Figure 1b). The inset in Figure 1b depicts an SEM-image of the nanochannel cross section and reveals a nearly rectangular shape with small features that are the consequence of the used etching process. These features do not impact the scattering properties of the channel, as we have corroborated in our earlier work by comparing an analytical model of cylindrical nanochannels with exact electrodynamic simulations of nanochannels with square cross sections.^51^ We also note that principally, the exact cross-sectional dimensions, as well as the nanochannel length, can be tailored within a wide range and according to the needs of a specific measurement since they are crafted using highly flexible micro- and nanolithography techniques described in detail in the Methods section. By connecting such nanofluidic channels to a microfluidic in- and outlet system described in more detail below, it becomes possible to flush liquid solutions^52,53^ but also gases^54^ through the channel and thereby change the RI difference between the solution in the channel and the surrounding medium, i.e., SiO_2_ in our current design. This change, in turn, alters the spectral distribution and intensity of visible light scattered from the nanochannel. Detecting these spectral changes constitutes the operation principle of the NSS.

### Theoretical Foundation of NSS

To describe the NSS principle on a more analytical basis, we approximate the rectangular nanochannel from the experiment as a cylinder of infinite length (Figure 1c). The validity of this approximation has been corroborated previously using finite-difference time-domain simulations.^51^ Furthermore, we adopt the description of the propagation of unpolarized incident electromagnetic waves and how they interact with a nanochannel from Bohren and Huffmann^55^ to arrive at an expression that describes the scattering cross section of a nanochannel, σ_channel_, as a function of the geometrical channel cross section, *A*_ϕ_, its (illuminated) length, *L*, and the wavenumber of the incident light, *k* = 2π/λ, as^46^
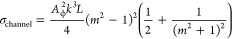
1

This expression also contains the parameter *m* = *n*_l_/*n*_SiO_2__, which is the ratio of the RIs of the liquid in the channel, *n*_l_, and of the surrounding medium of the channel, which is SiO_2_ in the present case, *n*_SiO_2__. It thus becomes evident that a change in *n*_l_ indeed induces a change in the nanochannel scattering cross section, provided all other variables remain constant.

As the first step to establish the fundamental understanding of the NSS methodology, it is relevant to analyze the wavelength-dependence of the RI of SiO_2_^48^ (channel matrix) and water^56^ (solvent used), as well as of their ratio, *m* (Figure 1d). Evidently, both RIs only weakly depend on the wavelength and in a very similar way such that *m* remains fairly constant across the visible wavelength range. Furthermore, calculating the scattering cross sections across the visible spectral range for a water-filled nanochannel for parallel (σ_p_), orthogonal (σ_o_) and unpolarized (average, σ_channel_) incident light reveals that its scattering cross section increases significantly for shorter wavelengths and most strongly for parallel polarization (see Figure 1d).

To illustrate how the optical contrast is generated in NSS, it is interesting to plot how the scattering cross section of the channel depends on *n*_l_ over a large *n*_l_-range for a given wavelength (here 600 nm, since it is the center wavelength of the spectrometer we use in our experiments, see Figure 5a), and again for parallel, orthogonal, and average polarization of the incident light (Figure 1e). A complete analysis for all wavelengths is given in the Supporting Information (Figure S2). This reveals that scattering vanishes when *n*_l_ = *n*_SiO_2__ and that it increases in an almost quadratic fashion when *n*_l_ ≤ *n*_SiO_2__, such that, e.g., an air-filled channel (*n* = 1) scatters much more light than a water-filled channel (*n* = 1.333). We also see that even very small RI changes induce a sizable change in the scattering cross section, as we have exploited in our previous work to quantify concentration changes induced by a catalytic reaction on single nanoparticles.^46^

For the reverse scenario, *n*_l_ ≥ *n*_SiO_2__, it is evident that the scattering cross section increases even more quickly per change of RI (Figure 1e). However, liquids with RI values larger than *n*_SiO_2__ are scarce and not commonly used as solvents. Nonetheless, this fact is interesting for future development since a nanofluidic system embedded into a matrix with a lower RI than the liquid inside it would boost the scattering intensity of the system and thus its ability to discern small RI contrasts.

Having established the theoretical foundation, it is now interesting to consider some first practical aspects. Specifically, we note that what is recorded in an NSS experiment is the spectrally resolved intensity of light scattered from a nanofluidic channel, that is, a scattering spectrum. In contrast, however, in traditional UV–vis spectroscopy, which we here call absorption spectrophotometry (ASP)—an absorption (or absorbance) spectrum is measured and enables the quantification of, e.g., molar extinction coefficients, which constitute a fundamental material constant. It is therefore of interest to introduce a general formalism, here first detached from nanochannels, that enables the mathematical transformation of an absorption spectrum into a scattering spectrum.

To do this, we first remind ourselves that the RI of a solid or liquid is a complex function of wavelength as
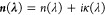
2

**Figure 2:**
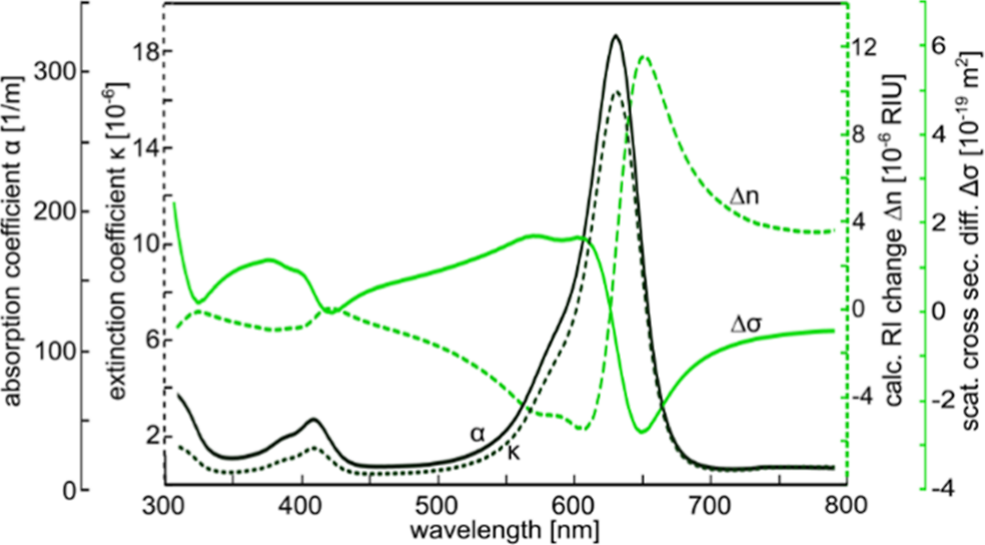
Analytical transformation from absorption to scattering spectrum for Brilliant Blue. We start with the spectrum of the absorption coefficient, α, for Brilliant Blue (black solid line) as obtained by ASP. In the first step, this spectrum is converted to the spectrum of the extinction coefficient, κ, (black dashed line). Subsequently, the corresponding change in RI spectrum, Δ*n* (green dashed line) is calculated using the Kramers–Kronig relation. Finally, the change in RI spectrum is converted to a scattering cross section spectrum difference, Δσ, of the nanochannel (green solid line). The corresponding transformations for Allura Red and Fluorescein are depicted in Figure S3.

Second, we consider the absorption coefficient spectrum, α(λ), using the Brilliant Blue dye (Figure 2, black solid line) measured by standard UV–vis spectroscopy as an example. It can be converted into an extinction coefficient spectrum, κ(λ) (Figure 2, black dashed line), using the expression
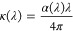
3Subsequently, since the extinction coefficient corresponds to the imaginary part of the RI of the dye (eq 2), we can calculate the spectral variation in the real part of the RI induced by absorption, Δ*n*(λ), (Figure 2, green dashed line) using the Kramers–Kronig relation^57−59^ as
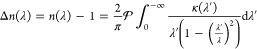
4

Finally, substituting the obtained expression for the wavelength-dependent RI, *n*(λ), of the dye into eq 1, we can connect the above analysis to the nanochannel framework. Accordingly, we can now calculate the expected change of the scattering cross section of a nanochannel, Δσ (Figure 2, green solid line), induced by a change of the liquid and thus the RI inside the channel, which may be induced by a change in the concentration of a solute or the complete exchange of said liquid.

### Nanofluidic Design

**Figure 3:**
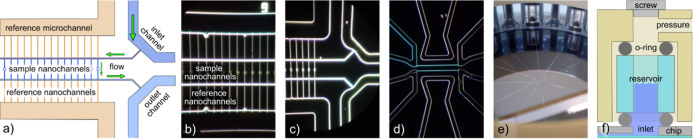
Nanofluidics layout. (a) Schematic layout of the two micro- and nanofluidic systems implemented on the chip. The reference nanochannel system used for online optical referencing (orange) has nanochannels that are arranged co-linearly with the sample nanochannels in the center (blue). Importantly, however, the two systems are not physically connected to ensure that the reference channel system always is filled with the desired reference liquid, i.e., the solvent (water in the present case), while the liquid is exchanged in the sample fluidic system during a measurement. Liquid access and exchange in the nanochannels are enabled via access microchannels (light blue for the sample system and orange for the reference system) by applying higher pressure to the inlet channel side. (b) Dark-field scattering microscopy image of the sample and reference nanochannels. The distance between the nanochannels is 20 μm and the sample channels are 62 μm long with a geometric cross section of 200 nm × 200 nm (cf. Figure 1b). The bright points in the center of the sample channels are constrictions that enable the trapping of colloidal nanocrystals, as we have demonstrated in earlier work.^45,46^ Their function is not used in this work. (c) Dark-field scattering image of the interface between micro- and nanofluidic systems. (d) Dark-field scattering image of the center of the fluidic chip at a lower magnification. Note the blue hue of the sample fluidics system as it has been filled with Brilliant Blue solution. (e) Photograph of the fluidic chip installed in the chip holder. The microfluidic systems are visible and connected to liquid reservoirs in the outer perimeter of the chip holder. (f) Schematic cross section of the inlet reservoirs showing how the chip holder connects to the chip.

For the NSS experiments, we designed a fluidic chip schematically depicted in Figure 3a and further illustrated by the corresponding dark-field scattering microcopy images taken at different magnifications (Figure 3b–d). As a key step beyond the state of the art,^45,46^ we have implemented two independent fluidic systems on the chip. The first one serves as the sample system (Figure 3a, blue) through which different sample solutions are introduced. The second one constitutes a reference system (Figure 3a, orange) filled with water at all times that we will use to compensate for, e.g., fluctuations in irradiated light intensity during a measurement. The sample solution exchange is enabled through a system of microchannels (50 μm wide and 1.2 μm deep), which at the inlet are connected to a macroscopic reservoir via an O-ring seal (Figure 3e,f), and which on the other end are connected to an array of parallel nanochannels with cross-sectional dimensions of 200 nm × 200 nm via a smaller microchannel (2 μm wide and 1.2 μm deep).

**Figure 4:**
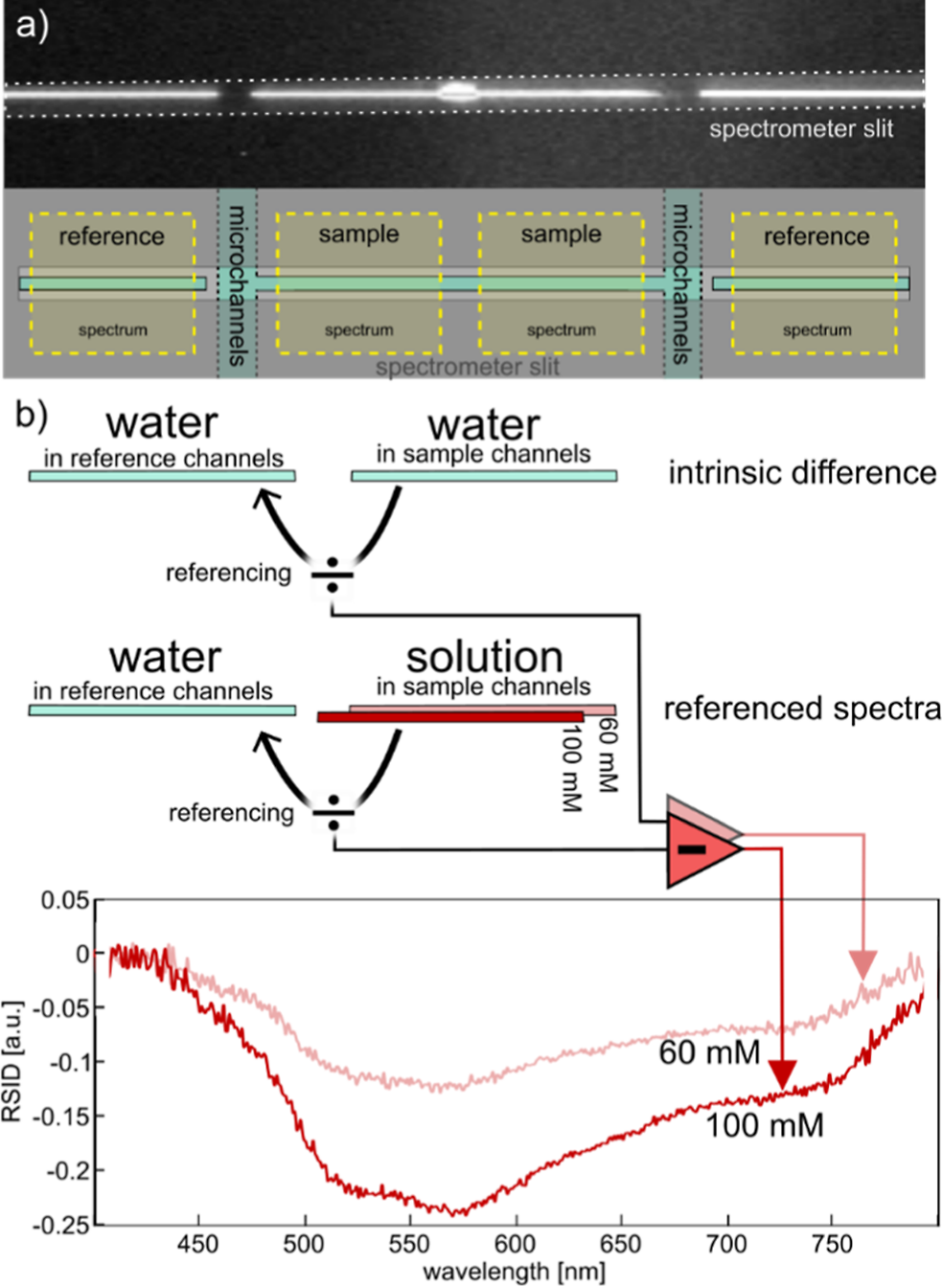
On-chip online optical referencing. (a) To enable the simultaneous online acquisition of sample and reference scattering spectra, we align a set of sample and reference nanochannels with the slit of the spectrometer. The nanochannels are sized such that spectra from two separate reference channels and from two separate areas of the sample channels can be recorded simultaneously (areas marked with yellow). The bright area in the center of the sample channel is again a constriction for colloidal particle trapping not used in this study. The microchannels are not visible due to the illuminating light being incident parallel to the microchannel walls. (b) Graphic depiction of the optical referencing scheme that delivers the RSID spectra: (i) both sample and reference nanochannels are filled with solvent (water) and the spectrum obtained from the sample channel is divided by the spectrum from the reference channel to deliver the intrinsic scattering intensity difference spectrum between sample and reference channel. (ii) The sample channel is filled with the solution of choice and the corresponding scattering spectrum from the sample channel is divided by the (constant) reference spectrum from the still water-filled reference channel. (iii) The intrinsic scattering intensity difference spectrum obtained in step (i) is subtracted from the normalized sample spectrum to obtain the RSID spectrum. (c) RISD spectra obtained by this procedure for 100 and 60 mM Allura Red solutions.

The same arrangement is mirrored on the outlet side of the chip. The reference nanochannels, which have the same cross-sectional dimensions as the sample channels to be optically identical, are arranged between and parallel to each of the sample channels, such that one sample and one reference channel fit within the opening of the slit of the spectrometer during an experiment (Figure 4g).

### Chip-Integrated Continuous Optical Referencing

The concept of continuous online optical referencing is a very efficient way to reduce noise and drift induced by fluctuating light intensities, change of focus of the microscope, or thermal (expansion) induced effects, and it is widely applied in dual-beam spectrophotometers. To implement such online referencing in an NSS experiment, we rely on the set of reference channels introduced above and the signal treatment sequence depicted in Figure 4a,b that comprises the following steps.

(i) Both the reference and sample channel systems are filled with water (or any other solvent used for a specific experiment) by applying a pressure of 2 bar to the corresponding reservoirs in the chip holder. A nanochannel pair is placed in the slit of the spectrometer, and four scattering spectra are recorded, two from the reference channel at upstream and downstream positions and two from the sample channel at the same upstream and downstream positions. It is here of critical importance that the illumination and observed channel length are as identical as possible for all positions because the signal ratio of sample and reference spectra during this step needs to be close to 1 for the evaluation scheme to be valid (see also Figure S4). One of the two sample-reference spectra pairs is used as a backup and control, as it may happen that the sample solution leaks into the reference channels or that the channels are compromised in another way. To record these spectra, we bin the signal from 50 pixels along the respective nanochannel and position, which corresponds to a ca. 30 μm long fraction of the respective nanochannel, to reduce the noise. Subsequently, we divide the obtained water-filled sample channel spectrum by the water-filled reference channel spectrum to obtain a what we call “intrinsic difference spectrum” between sample and reference channel. We will use this intrinsic difference spectrum (examples shown in Figure S4a) in the last analysis step (iii) to account for the intrinsic differences in scattering profile that (may) exist between a reference and sample channel when they are filled with the same liquid, e.g., due to slightly different dimensions or surface roughness. (ii) The water in the sample channel system is exchanged by an aqueous solution of the compound of interest (here the dye Allura Red, as a first example), by exchanging the liquid in the corresponding reservoir and again applying 2 bar of pressure to establish a flow through the nanochannel. The measured scattering spectrum from the sample channel is then divided by the simultaneously obtained spectrum of the water-filled reference channel. (iii) As a final step, the intrinsic difference spectrum measured in step (i) is subtracted from the referenced sample spectrum obtained in step (ii), resulting in what we call a “relative scattering intensity difference” (RSID) spectrum.

### NSS Measurements of Nonabsorbing Solutes

**Figure 5:**
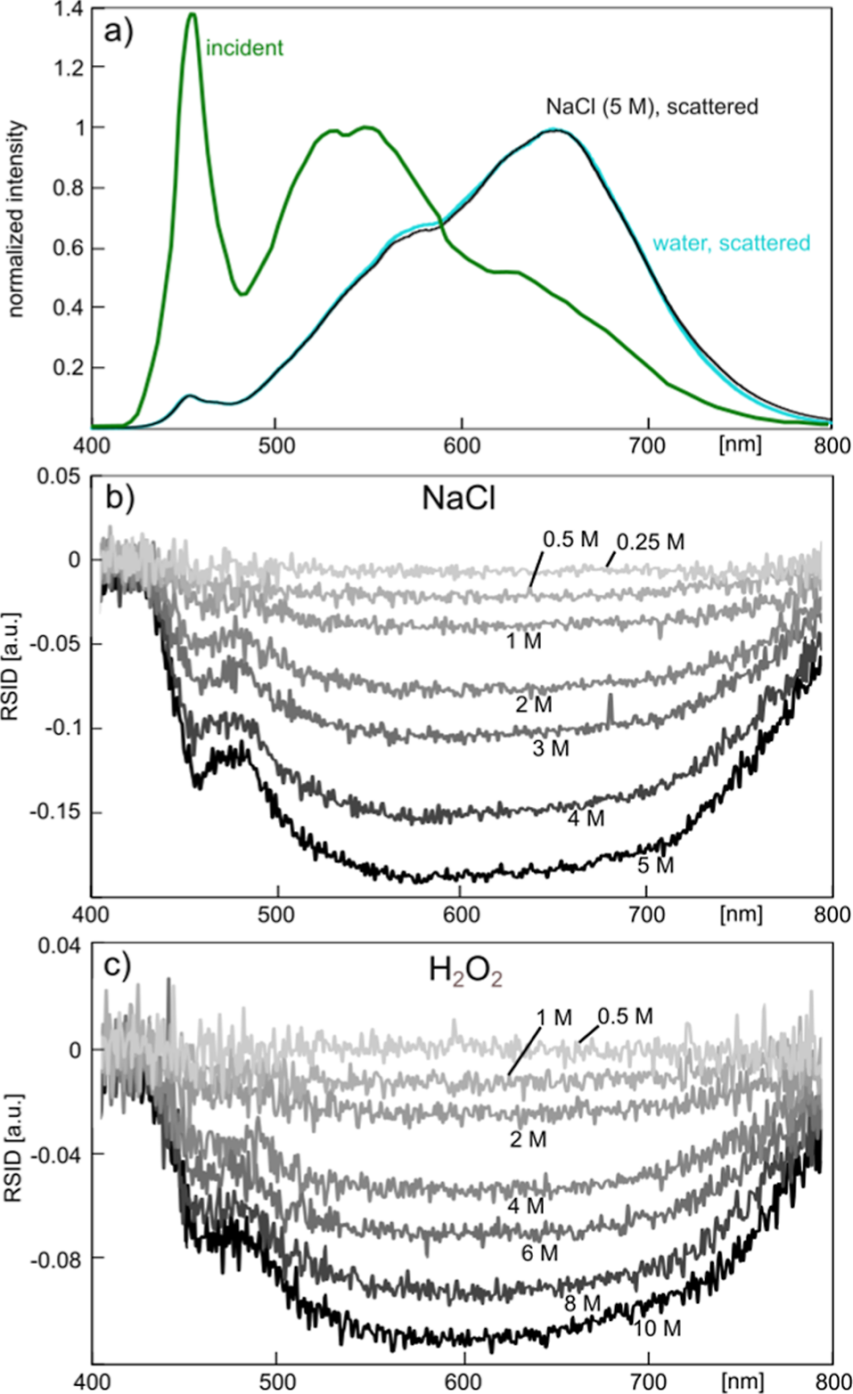
NSS of NaCl and H_2_O_2_ solutions. (a) Incident emission intensity spectrum of the LED light source prior to entering the microscope (green), this light scattered from a water-filled nanochannel measured through the NSS microscope setup (cyan), and this light scattered from a nanochannel filled with a 5 M NaCl solution measured through the NSS microscope setup (black). All spectra have been normalized to their maximum value in the wavelength range between 500 and 700 nm. Relative scattering intensity difference (RSID) spectra for (b) NaCl and (c) H_2_O_2_ at different concentrations in water, as indicated by the labels.

To illustrate the principle of NSS and its application as a method for the detection of solute concentrations inside a nanofluidic channel, we consider two solutes that are transparent, i.e., do not absorb light in the UV–vis regime: NaCl and H_2_O_2_ were dissolved in water (Figure 5). To establish these experiments, it is illustrative to first discuss the irradiated spectrum produced by the used LED white light source and how it is affected by the optical elements in the experimental setup comprising a microscope, spectrometer, and CCD camera, to understand the origin of specific features in the spectra obtained in our experiments. The light emitted from the LED has a relatively broad emission band spanning from 420 to 760 nm, with the highest intensity at around 540 nm. In addition, there is a strong peak at 450 nm (Figure 5a). However, when this light is scattered from a water-filled nanochannel, the maximum intensity of the scattered spectrum is significantly shifted when measured through our microscope system (Figures 5a and S5). This is the consequence of (i) the spectral sensitivity of the CCD camera, (ii) the wavelength-dependent efficiency of the used grating in the spectrometer, and (iii) the transmittance characteristics of other optical elements on the microscope, such as the objective (Figure S1). Together, they skew the emission spectrum of the lamp to longer wavelengths and thus, e.g., strongly decrease the 450 nm peak in the irradiated spectrum. However, when exchanging the water in the sample channels to a 5 M NaCl solution, the overall shape of the scattered light spectrum obtained from a sample volume of about 1.2 fL (corresponds to the 30 μm long nanochannel section used) remains globally very similar since there are no absorption bands for the NaCl solute (Figure 5a). Nonetheless, we notice sizable differences in scattered light intensity that are most pronounced around 600 nm, which we can ascribe to the presence of the solute and the corresponding change in RI of the solution.

As the next step, we extracted RSID spectra for seven different NaCl concentrations ranging from 0.25 to 5 M in water, according to the procedure introduced in the previous section (Figure 5b). Due to the lack of absorption bands of this solute, the obtained RSID spectra are broad and featureless, with a strictly negative amplitude due to an increasing RI compared to pure water upon increasing solute concentration. This amplitude is proportional to the solute concentration, as further quantified below. Similar results are obtained when using H_2_O_2_ as the solute, however, with sizably different RISD (Figure 5c). These measurements demonstrate that concentration-dependent UV–vis scattering spectra of a nonabsorbing solute can be obtained from 1.2 femtoliter sample. If we consider here the highest concentration of each solute, the number of molecules sampled in Figure 5b,c is 3.6 × 10^9^ for NaCl and 7.2 × 10^9^ for H_2_O_2_, and for the lowest concentration, it is 1.8 × 10^8^ and 3.6 × 10^8^, respectively.

### NSS Measurements of Light-Absorbing and Fluorescent Dyes

Since traditional UV–vis spectroscopy is widely applied to determine solute concentrations, as well as characteristic fingerprints of molecules that exhibit absorption bands in the UV–vis spectral range, we in the next step apply NSS to a selection of organic and fluorescent dyes, i.e., Brilliant Blue, Allura Red, and Fluorescein, in the concentration regime from 5 to 100 mM.

**Figure 6:**
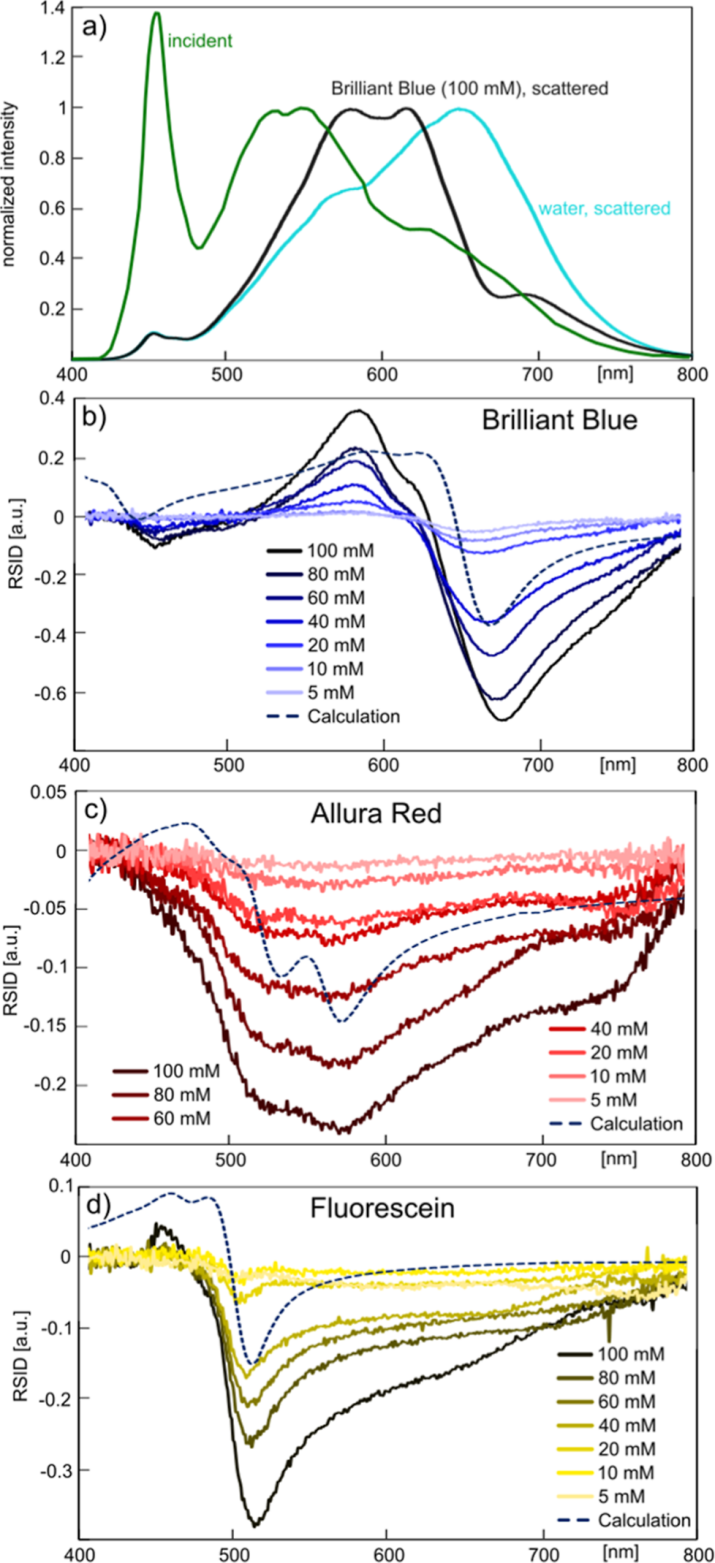
NSS of Brilliant Blue, Allura Red, and Fluorescein solutions. (a) Incident emission intensity spectrum of the LED light source measured directly, i.e., not through the microscope setup (green), this light scattered from a water-filled nanochannel measured through the NSS microscope setup (cyan), and this light scattered from a nanochannel filled with a 100 mM Brilliant Blue solution measured through the NSS microscope setup (black). All spectra have been normalized to their maximum value in the wavelength range between 500 and 700 nm. RSID spectra for (b) Brilliant Blue, (c) Allura Red, and (d) Fluorescein at different concentrations in water, as indicated by the legend. Each panel also includes a scaled back-calculated scattering spectrum obtained from ASP measurements of the respective dye solution.

Starting again at the level of the raw measured scattering spectra and taking a 100 mM Brilliant Blue solution as the example, we notice that the distinct absorption bands of the dye are clearly reflected when comparing the spectra obtained from a water-filled and a dye-filled nanochannel (Figure 6a). Subsequently deriving the RSID spectra for the 1.2 fL sample volume (30 μm long nanochannel section) for the entire concentration range reveals a systematic dependence of RSID intensity on concentration as well as a distinct peak at 580 nm with a weaker “shoulder” at 630 nm and a distinct negative peak at 680 nm (Figure 6b). These features correspond to rising and falling flanks in the absorption spectra of the dye (cf. Figures 2 and S3) and can be reasonably reproduced when calculating theoretically the expected scattering spectrum of Brilliant Blue inside a nanochannel using the formalism derived above, i.e., eq 4 (dashed line in Figure 6b, see also Figure S3). The observed discrepancies between calculated and measured RSID spectra are most likely the consequence of the significant spectral modulation of the irradiated light intensity in our setup (Figure 5a and corresponding discussion), which enhances or suppresses certain features relative to each other, depending on their spectral position. As a last point, we notice that for Brilliant Blue, both positive and negative RSID amplitude changes can be observed because of positive or negative RI-differences induced by the different absorption bands compared to pure water.

Executing the same analysis for the dye Allura Red (Figure 6c) and the fluorescent dye Fluorescein (Figure 6d) in the same concentration range and the 1.2 fL sample volume generates overall very similar results with reasonable agreement between calculated (see Figure S3) and measured RSID spectra and with distinct concentration dependence of the RSID signal amplitudes. We note that we do not resolve the slight positive RSID peak for Allura Red predicted by the calculated spectrum. This is the consequence of the dye’s small extinction coefficient and the low transmittance of our microscope setup in the wavelength range where the peak is expected to occur, as evident from Figure 6a. We also note that for Fluorescein, we do not resolve a scattering band that would correspond to the 540 nm fluorescence emission line. The likely reason is that the number of emitted fluorescence photons is much lower than the number of scattered photons from the nanochannel since the number of fluorescein molecules in the channel at the given concentrations is very low, i.e., on the order of 10^7^ molecules.

**Figure 7:**
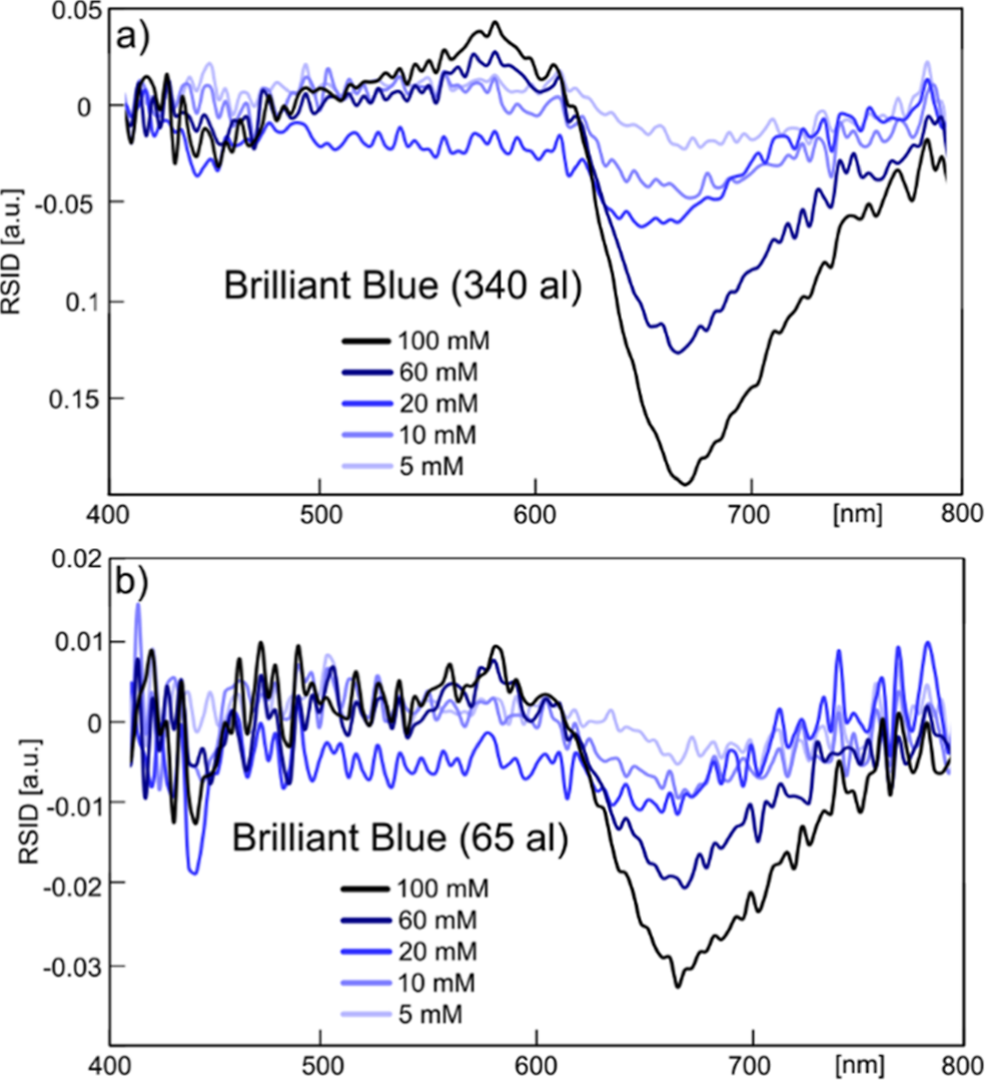
NSS from attoliter sample volumes. (a) RSID spectra for different concentrations of Brilliant Blue in water taken from a 19 μm long section of a 100 nm × 180 nm channel, resulting in a sample volume of 340 aL. (b) Same as (a) but here spectra were taken from an only 3.6 μm long section of the nanochannel, reducing the sample volume to 65 aL.

To put this number into perspective and compare NSS in this respect to typical solute concentrations and sample volumes used in ASP, we recall that in standard ASP cuvettes, with an optical path of 1 cm through the sample solution, the commonly used solute concentrations range from 1 to 10 μM (the range of concentrations used for the ASP dye spectra in Figure S3, see also our previous work^49^). Hence, assuming an irradiated light beam cross section of 1 cm^2^, the sample volume probed by the beam corresponds to 1 cm^3^. With the given solute concentration range of 1–10 μM, this means that between 3 × 10^15^ and 3 × 10^16^ molecules are sampled in a typical ASP measurement of a light-absorbing solute. In contrast, for NSS, we operate in the 100 mM concentration range but with a sample volume of 1.2 fL (or ca. 8 × 10^–13^ cm^3^) only. This translates to 72 × 10^6^ molecules probed in an NSS experiment at 100 mM concentration and corresponds to a staggering 9 orders of magnitude reduction in the number of molecules required. This, in combination with the fact that the NSS sample volume can be further reduced to the attoliter (aL) regime by reducing the nanochannel dimensions and/or the channel section used for analysis, highlights the ability of NSS to work with very tiny amounts of sample substance. As proof for this point, we fabricated a fluidic system identical in structure to the one described above but now featuring sample nanochannels of 100 nm width and 180 nm depth with a total length of 120 μm. By then splitting the total length of the channel into longer (19 μm) and shorter (3.6 μm) segments and using Brilliant Blue dye across the 5 to 100 mM concentration regime, we reduced the sample volume from which the RSID spectra are recorded to 340 aL (20.5 × 10^6^ molecules @ 100 mM and 1.025 × 10^6^ molecules @ 5 mM, Figure 7a) and 65 aL (4 × 10^6^ molecules @ 100 mM and 2 × 10^5^ molecules at 5 mM, Figure 7b), respectively. Clearly, the spectral fingerprint of the dye is still resolved, as is the case for these tiny sample volumes. A comparison with the RSID spectra taken for the same dye concentrations in the larger nanochannels (cf. Figure 6b) reveals that the positions of the spectral features are identical, even though noise level increases and absolute RSID is decreased due to the smaller scattering cross section of the smaller channel and the smaller number of molecules inside said smaller channel (Figure S6). Taken all together, these results corroborate the ability of NSS to resolve the distinct spectral fingerprints of dye molecules in the visible spectral for sample volumes in the attoliter to femtoliter range, for concentrations in the 5 to 100 mM concentration regime. This corresponds to as few as 2 × 10^5^ probed molecules only, which is 10 orders of magnitude fewer molecules than usually probed in the established ASP.

### Concentration Dependence

**Figure 8:**
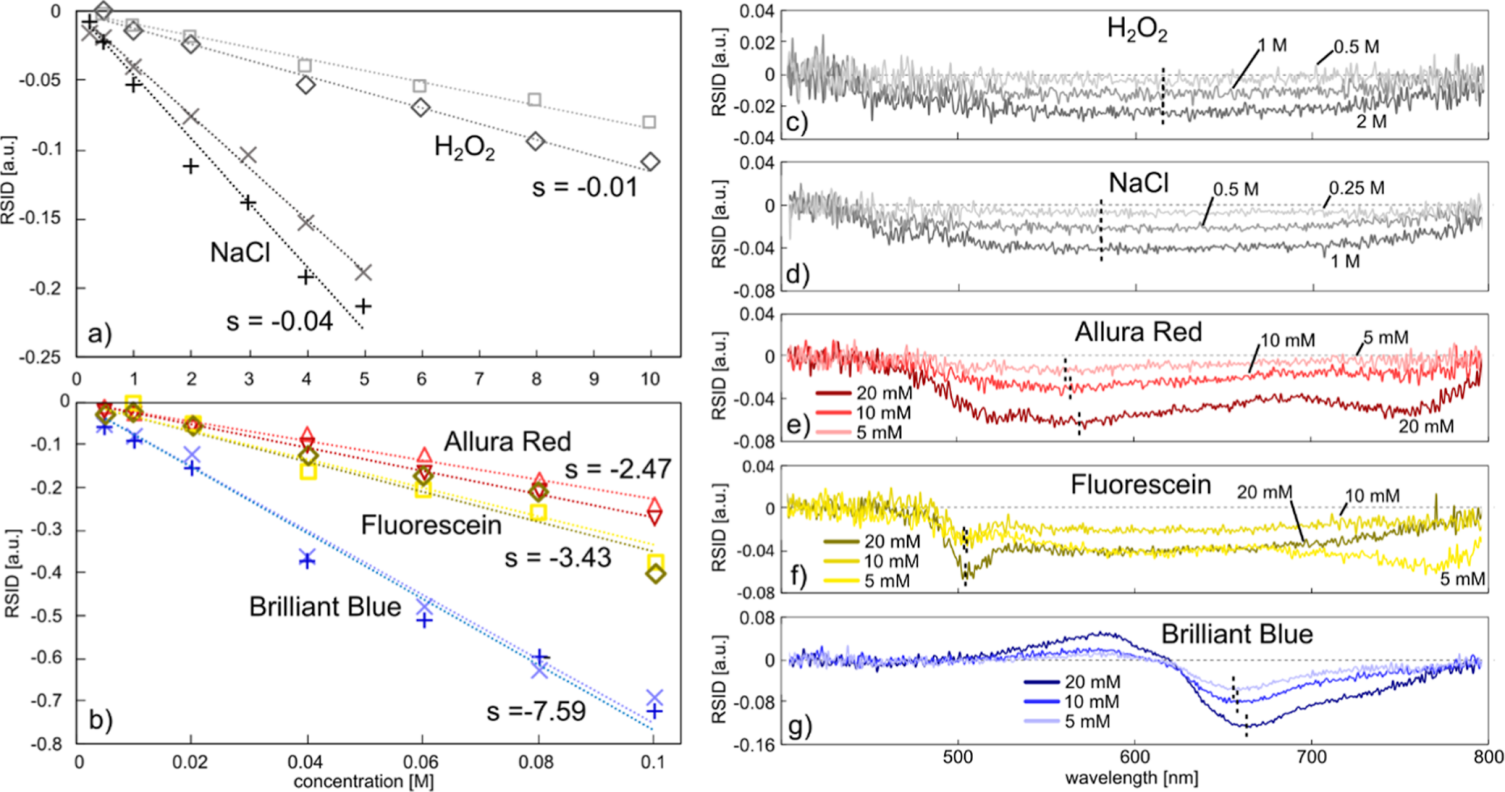
Solute concentration dependence. (a) Comparison of the colorless solutes H_2_O_2_ and NaCl in terms of RSID change per change in concentration of the solute in the nanochannel for two independent measurement series. The symbols are data points taken at 580 nm (for H_2_O_2_) and 612 nm (for NaCl) where the RSID contrast is largest. The dashed lines are linear fits to the data points whose slope, *s*, is indicated as average of the two respective measurements. (b) Same as (a) but for the dyes Allura Red (measured at 570 nm), Brilliant Blue (measured at 665 nm), and Fluorescein (measured at 510 nm). The respective peak positions given were used for all dye concentrations above 20 mM, where an average of 10 data points around this position was taken. For concentrations lower than that, the peak positions seemed slightly shifted, so that the peak position for the evaluation was adapted [vertical dashed lines in (c–g)]. (c–g) The RSID spectra for the three lowest concentrations of each solute together with the peak position (black dashed lines) used for extracting the data points in (a,b).

To further develop the NSS concept with a focus on quantitative information that can be extracted, we focus on the concentration dependence of NSS spectra. As mentioned above, the proportionality between the RSID amplitude and the RI of the liquid in the channel (eqs 1 and S1) enables the quantitative determination of the concentration of a specific solute in a nanochannel. To further explore this opportunity, we plot the RSID amplitude extracted at the wavelength with the strongest response for each compound vs the predetermined concentration for the five solutes investigated in this study (Figure 8a,b), i.e., H_2_O_2_ (RISD at 612 nm, Figure 8c), NaCl (RISD at 580 nm, Figure 8d), Allura red (RISD at 570 nm, Figure 8e), Fluorescein (RISD at 510 nm, Figure 8f), and Brilliant Blue (RISD at 665 nm, Figure 8g), again using the larger channels with the 1.2 fL sample volume.

The first key observation is that all data points across the 5 different solutes follow a linear relation to a good first approximation since the range of RI change at hand is sufficiently small. This is in good agreement with the prediction in Figure 1e. Practically, this means that an NSS fluidic chip can be precalibrated for concentration measurements of a specific solute and thus enable accurate measurements of the concentration dependence of solutes in sample volumes in the femtoliter regime and below.

The second key observation is that each solute has a specific slope for this linear relation, which corresponds to the change in the scattering intensity per change in the concentration at the given wavelength. We find the smallest slope for H_2_O_2_ (−0.01 RSID/M) and the largest one for Brilliant Blue (−7.59 RSID/M), as shown in Figure 8a,b. The underlying reason for the different slopes exhibited by different compounds is straightforward to understand for NaCl and H_2_O_2_, since they do not absorb light in the visible regime. Hence, it is only the actual change of the real part of the RI that causes the change in RSID. At an absolute scale, the difference in slope between H_2_O_2_ (−0.01 RSID/M) and NaCl (−0.04 RSID/M) can be explained by considering the Lorentz–Lorenz formula that directly connects RI and the polarizability of the solutes.^60^ To this end, in their investigation of the RI of salt water, Aly and Esmail^60^ concluded that the RI of a solution is determined by the ratio of solute and solvent molecules but also by the molar polarizability of each species. Hence, based on the different slopes depicted in Figure 8a, it is clear that the molecular polarizability of NaCl in water is higher than that of H_2_O_2_. The reason is the dissociation of NaCl into its charged ionic constituents, while H_2_O_2_ remains molecularly intact, which makes the dissolved Na^+^ and Cl^–^ ions more responsive to the external electrical field of light.

Turning to the dyes, which all exhibit strong absorption bands in the visible spectral range, the observed larger slopes have their origin in the change in RI caused by the strong absorption in the visible regime. This means that the slopes depicted in Figure 8b are directly connected to the molar extinction coefficient, ε, for each solute, as shown in Table 1.

**Table 1:** Comparison of RISD Concentration Slopes and the Molar Extinction Coefficient, ε, for the Allura Red and Brilliant Blue Dyes and for Fluorescein

dye	RSID slope [RSID/M]	ε [10^4^ M^–1^ cm^–1^]
Allura Red	–2.47	2.78^61^
Fluorescein	–3.43	7.69^62,63,64^
Brilliant Blue	–7.59	13.0^65^

This clear connection between change in scattering intensity and molar extinction further illustrates how compounds can be identified and quantified at the nanoscale using NSS, since a solute with a higher molar extinction coefficient will have a higher amplitude in the RSID spectrum at the same concentration.

The second aspect of relevance here is the lowest concentrations of a specific solute that can be resolved. It becomes clear from the RSID spectra of our five solutes taken at the three lowest concentrations, for each respective system in a 200 nm deep and wide nanochannel, that for the two nonabsorbing solutes, we are approaching the limit of detection in the 250–500 mM range (Figure 8c,d), whereas for the dyes, the limit is roughly one order lower, i.e., in the low mM range or below (Figure 8e–g). If desired, either increasing the intensity of the irradiated light^51^ or increasing the nanochannel cross section, since the scattered intensity is proportional to channel dimensions (see eq 1), will enable measurements at lower concentrations.

### Extracting Molar Extinction Coefficients from NSS Spectra

**Figure 9:**
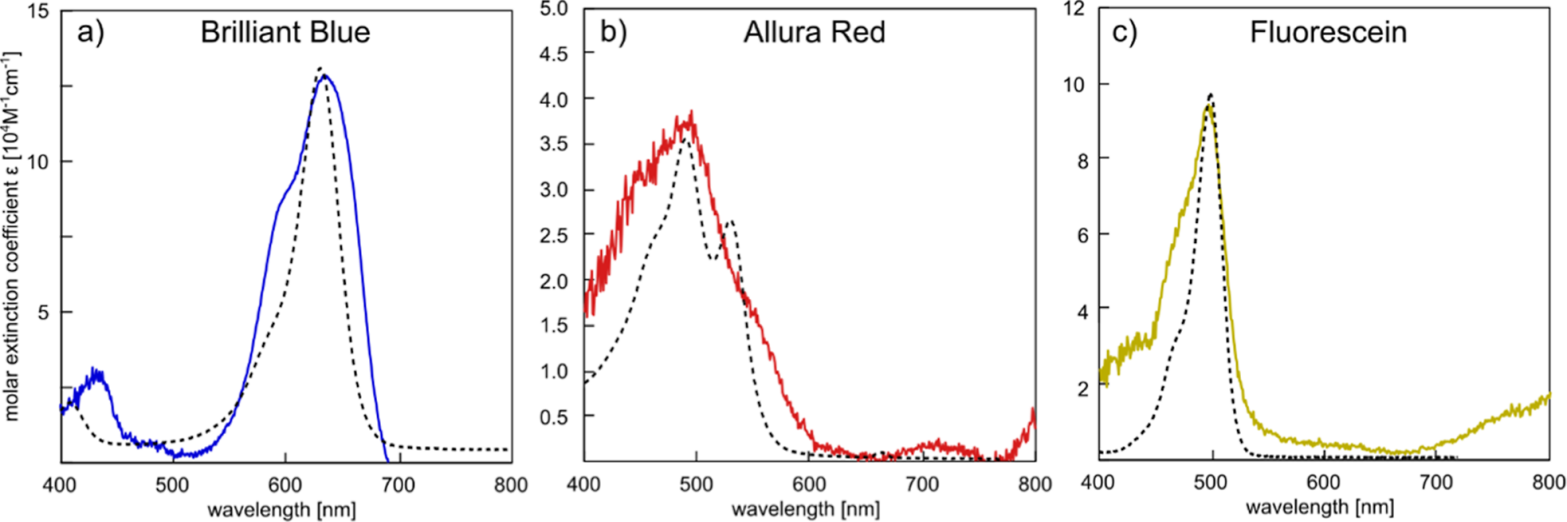
Molar extinction coefficient spectra obtained by ASP and NSS. Molecular extinction coefficient spectra for (a) Brilliant Blue, (b) Allura Red, and (c) Fluorescein, as measured using traditional ASP (3.8, 14 and 19 μM concentration, respectively—dashed lines) and obtained by NSS (solid lines) via the back-calculation from RISD spectra detailed in Supporting Information Section SII.

Having established the ability of NSS to deliver quantitative information about solute concentration in the molar to millimolar concentration range in femtoliter and attoliter sample volumes, as the last aspect, we explore the possibility of determining the molar extinction coefficients of the solutes. To do this, we first reconnect to the beginning of this work, where we used the Kramers–Kronig relation (eq 4) to analytically link the change in optical absorption of a solution to a corresponding change in its RI, which in turn is the reason for the scattering intensity difference measured by NSS. Using the same principle, it is possible to calculate a molar extinction spectrum from an RSID spectrum (which reflects the underlying RI spectrum) produced by NSS. The detailed analytical derivation of the mathematical formalism is presented in Supporting Information Section II. In brief, the reverse calculation of molar extinction coefficients from RSID spectra consist of the following steps: (i) recalling that RSID is the ratio of the sample and reference channel scattering intensities, which means that it also is the ratio of the scattering cross sections of the sample and reference nanochannels (see eq S1). (ii) Using eq 1 to relate the scattering cross section of the nanochannels to the RIs of water (the solvent here), the solution itself, and the material the nanochannel is embedded in, i.e., SiO_2_. (iii) Calculating the RI spectrum of the solution and fitting a Cauchy-type curve to the obtained RI spectrum to enable subsequent subtraction of this “normal” dispersion from the “anomalous” dispersion, i.e., here the absorption bands of the dye that we are interested in since they eventually translate into the extinction coefficient. To this end, the Cauchy formula is a basic analytical description of the dispersion in a transparent medium,^66^ whose simplicity facilitates the separation of “normal” and “anomalous” dispersion in a reasonably straightforward way. We note that for cases where the investigated spectral range extends further into NIR-regime, a fit of the Sellmeier-equation^67^ may be more appropriate. (iv) Using the isolated change in the RI spectrum that stems from anomalous dispersion by the dye absorption bands and applying the Kramers–Kronig formula to calculate an extinction coefficient spectrum. (v) Calculating the molar extinction coefficient spectrum based on the known concentration of the solute in the solution. This calculation is relatively straightforward, since all geometry-dependent factors can be canceled out because reference and sample nanochannels have identical dimensions. However, we note that the quality of the Cauchy-fit to the RI spectrum is important when the molar extinction coefficient spectrum is to be calculated from scattering, since the presence of the dye molecules alone (irrespective of their specific absorption bands) changes the RI of the solution, as shown for the nonabsorbing solutes NaCl and H_2_O_2_. The fit therefore needs to include all contributions to the RI that do not stem from anomalous dispersion such that they can be subtracted. However, this subtraction is challenging in practice, since the exact contribution of normal and anomalous dispersion is not known. Hence, the applied Cauchy-fit is to be regarded as a reasonable first approximation to the problem at hand, whose validity is corroborated by the good agreement we obtain in terms of extracted extinction coefficients for all three dyes (Figures 9 and S7). We also note that accurately knowing the RI of the surrounding material (a Borofloat 33 cover glass bonded to the thermal oxide of the silicon wafer in our case) is equally important, since it directly determines the amplitude of the RSID spectra. This becomes evident from eq 1, which states that the ratio of the RI of the channel content and the RI of the channel embedding matrix contribute almost quadratically, making NSS as sensitive to the RI outside of the channel as to the RI inside it.

With these minor reservations, we now apply this formalism to the three dyes to calculate their molar extinction coefficient spectra from the RSID spectra and compare them with the ones obtained from traditional ASP (Figure 9a–c). Overall, we find very good agreement for all three dyes. For a more detailed inspection, we first consider the spectra for Brilliant Blue and see that both the main absorption peak at 630 nm and the shoulder at 590 nm match well in the NSS and ASP spectra (Figure 9a). In particular, the agreement in peak amplitude at 630 nm, and thus in the absolute molar extinction coefficient for the main dye absorption band, is excellent. Furthermore, we find that the 590 nm shoulder is more pronounced in NSS when compared to the ASP spectrum, which we attribute to the specifics of the illumination spectrum in the NSS experiments that exhibits a peak at this position (cf. Figure 6a). The small peaks below 450 nm in Figure 9a appear offset by about 20 nm. As a result, we argue that the peak at 450 nm in the NSS spectrum first and foremost stems from the peak in the irradiated light spectrum (cf. Figure 6a) since it overlaps with actual absorption peak of the dye at 412 nm. This convolution of two contributions renders the peak in the NSS extinction coefficient spectrum slightly skewed to shorter wavelengths, which is the reason for the observed apparent offset.

Turning to Allura Red, we find the main absorption peak with both methods at 490 nm and with excellent agreement in terms of maximum amplitude and thus molar extinction coefficient for the main absorption band (Figure 9b). The second peak at 540 nm, distinctly resolved by ASP, is less pronounced in the NSS spectrum. We attribute this primarily to the decreased scattering intensity in this area (cf. Figure 6a), but it could also be that the Cauchy-fit is distorting the resulting spectrum slightly since the exact contribution of the anomalous dispersion to the RI spectrum is unknown, as discussed above. For Fluorescein (Figure 9c), the position of the main peak at 500 nm, as well as its amplitude, are in almost perfect agreement for NSS and ASP. The amplitude of the shoulder on the short-wavelength-side of the main peak is slightly out of proportion, again due to the low light transmission through the microscope in this spectral range and the corresponding reduction of the S/N.

As the last aspect, we briefly mention possible improvements to NSS as a method for the measurement of the molar extinction coefficient of solutes: (i) more exact measurements of the RI of the matrix material that surrounds the nanochannel; (ii) optimization of the fit to the contribution of the normal dispersion to the RI spectrum by, for example, using a polynomial instead of a Cauchy curve; (iii) reduction of the general noise level in the data by increasing the exposure time, the number of averaged spectra, and the intensity of the incident light, or by using a camera with lower background noise for recording; (iv) optimizing the microscope setup and spectrometer grating to enable higher light transmission at shorter wavelengths to increase S/N in this range, and thereby better resolve spectral features in this regime.

## Conclusions

We have introduced nanofluidic scattering spectroscopy, NSS, which measures visible light scattered from a single nanofluidic channel in a spectrally resolved way, as a tool for the spectroscopic investigation of colored and transparent solutions inside nanofluidic channels in continuous flow fashion and for sample volumes as small as a few tens of attoliters in the millimolar concentration regime, which corresponds to as few as 10^5^ probed molecules. As a further key step beyond the state of the art, we have implemented two independent fluidic systems on the used chip. The first one served as the sample system through which different sample solutions were introduced, whereas the second one constituted an optical reference system filled with water at all times, which we used to compensate for, e.g., fluctuations in irradiated light intensity during a measurement. In this way, we were able to implement a similar concept on a nanofluidic chip as widely used in dual-beam spectrophotometers to establish long-term stable measurement conditions. On the examples of the nonabsorbing solutes NaCl and H_2_O_2_, and the dyes Brilliant Blue, Allura Red, and Fluorescein, we subsequently demonstrated that their spectral fingerprints can be obtained with good accuracy and that solute concentrations inside a single nanochannel can be determined based on NSS-spectra from femto- to attoliter sample volumes. Furthermore, by applying a reverse Kramers–Kronig transformation to measured NSS-spectra of solutes inside a single nanochannel, we demonstrated that the molar extinction coefficient spectrum of the solute can be extracted with very good agreement with literature values, thereby enabling the identification of solutes based on a fundamental material constant.

Looking forward, our findings advertise NSS as a versatile tool for the spectroscopic analysis of solutes in situations where nanoscopic sample volumes and continuous flow measurements are of importance. An example for such a situation is single particle catalysis inside nanofluidic channels, which has the aim to resolve catalytic conversion on the surface of a nanoparticle localized inside the nanochannel by preventing excessive dilution of the reactant products due to the tiny volume of the said nanochannel^45,49,54,68^ To this end, for this application, we have already demonstrated the key importance of limiting the detection to a fraction of a nanochannel^46^ and argue that adding the spectroscopic dimension enabled by NSS further leverages the potential of single particle catalysis in nanofluidic systems as a whole. Furthermore, since it is possible to realize various concentration, temperature, and pressure scenarios inside nanofluidic systems, we envision that the NSS concept also may find application in homogeneous catalysis, where, e.g., the absorption of photosensitizers^69^ could then be investigated on only fractions of the usual sample volume, or in biological sample sequencing and medical drug development where costly molecules initially are available in tiny amounts and volumes only. To reduce the total sample volume required in such experiments, i.e., the volume equivalent to the entire fluidic system connecting to the nanochannels and not only the nanochannels themselves, the micro- and nanofluidic concepts employed offer ample opportunities as they easily can be downscaled in their entirety.

## Methods

### Instruments

The exchange and flow of solutions in/through the nanofluidic chip was realized by applying pressure to the inlet reservoirs of the chip holder with a Fluigent MFCS-EX pressure controller. The dark-field microscopy images were taken on a Nikon Eclipse LV150N upright microscope equipped with a Nikon 50× ELWD dark-field objective. Illumination was provided by a Thorlabs Solis-3C LED light source. The light scattered from the nanochannels was directed into an Andor spectrometer (SR-193I-A-SL) that had a 150 L/mm grating installed. The spectrally resolved light was recorded with an Andor Newton (DU920P-BEX2-DD) camera attached to the spectrometer, which binned the four areas (upper and lower reference channels and upper and lower signal channels) to a total of four spectra, which were used for subsequent data evaluation. The spectra were recorded with 2 s of exposure time and consisted of 10 accumulated pictures. For Figures 5 and 6, additional spectra of the incident light were recorded using an Avantes AvaSpec-1024 fiber spectrometer. ASP spectra of the dyes were recorded on a Varian Cary 50 Bio UV–vis spectrophotometer. The SEM images of the channel and its cross section were recorded on a Zeiss Supra 55VP scanning electron microscope.

### Sample Preparation

All dyes (Allura Red, Brilliant Blue, Fluorescein) were bought from Merck as solid material and diluted into the required concentrations by mixing the dyes with ultrapure water (Milli-Q IQ 7000 water purification, Merck). A similar procedure was carried out for NaCl and H_2_O_2_, where the latter was acquired as a solution (H_2_O_2_, 35% w/w in H_2_O, Merck). Injection into the fluidic chip holder was done with syringes and blunt needles (Braun).

### Fluidic Chip Fabrication

The micro- and nanofluidic chips used in the experiments were fabricated in the clean room facilities of MC2 at Chalmers in Gothenburg. Each chip consisted of a single 4 in. silicon wafer with a thermal oxide layer into which the fluidic structures were etched. The general procedure is described in detail in our earlier work by Levin et al.^49^ As a short summary: The 4 in. (100) silicon wafers were cleaned with Standard Clean 1, followed by a 2% HF bath and Standard Clean 2. Growing of the thermal oxide layer was carried out in a wet atmosphere at 1050 °C until a thickness of 250 nm was reached. Etching of the nanochannels was done via fluorine-based reactive ion etching after they were patterned in a resist layer by electron beam lithography. Subsequently, the microchannels were etched into the thermal oxide and Si substrate by the same method, and the photoresist was patterned here with direct laser lithography. As a final step, the substrates were cleaned with Standard Clean 1, together with a 175 μm thick Borofloat 33 glass wafer to be used as a lid of the fluidic system. This glass lid was equipped with inlet holes (sandblasted) to match the liquid reservoir on the nanofabricated wafer. The surfaces of both wafers were then treated with O_2_ plasma (50 W, 250 mTorr) to enable prebonding of the glass cover lid to the wafer with the fluidics. The subsequent fusion bonding was carried out at 500 °C for 5 h. After bonding the 4 in. fluidic wafer was cut into shape to fit the chip holder.

## Data Availability

The underlying data for this publication are available at Zenodo, 10.5281/zenodo.10848089.

## Supporting Information Available

Schematic of the experimental setup, nanochannel scattering cross section dependence on wavelength and RI, spectra of Brilliant Blue, Allura Red, and Fluorescein during the analytical transformation from absorption spectra to scattering spectra, intrinsic scattering spectra and evaluation strategies, spectra of the light used during the experiment, comparison of spectra from different channel sizes, and calculation of the molar extinction coefficient from measured RSID spectra (PDF)
