# Supplementary material for: Visible Light Spectroscopy of Liquid Solutes from Femto- to Attoliter Volumes Inside a Single Nanofluidic Channel
Source: ACS Nano. 2025 Jan 7;19(2):2857–69. doi: 10.1021/acsnano.4c15878 (PMC11760169; doi:10.1021/acsnano.4c15878)
Supplement: Supplementary file 1 — nn4c15878_si_001.pdf [file nn4c15878_si_001.pdf]

Supplementary Material for

Visible Light Spectroscopy of Liquid Solutes from Femto- to Attoliter Volumes inside a Single Nanofluidic Channel

Björn Altenburger[1], Joachim Fritzsche[1] and Christoph Langhammer[1]*

[1]Department of Physics, Chalmers University of Technology; SE-412 96 Gothenburg, Sweden

*Corresponding author: clangham@chalmers.se

Section I: Supplementary figures

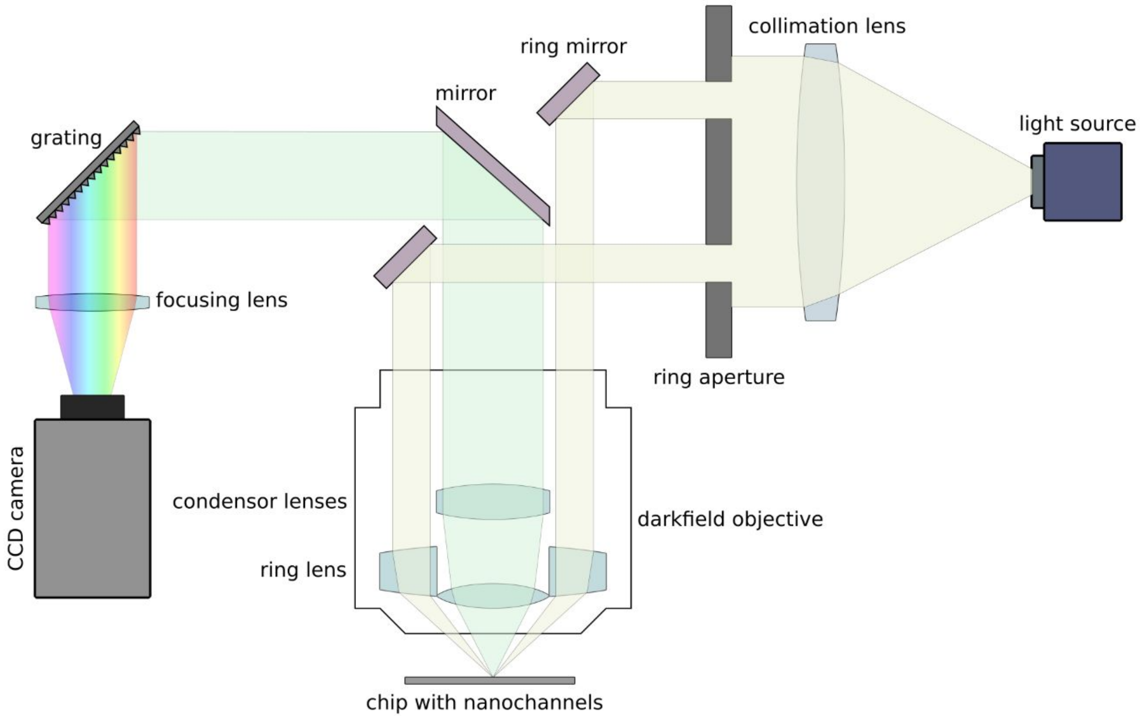

Figure S1. ***Schematic of the experimental setup.*** *The illuminating polychromatic white light is emitted by a broad-spectrum LED-lamp (Thorlabs Solis 3C) and collimated into the microscope (Nikon Eclipse LV150N). Here, it is partly blocked by a ring aperture that transmits only a ring-shaped section of the light onto a ring-shaped mirror, from where it is directed into the dark-field objective (TU Plan ELWD 50x/0.6 B OFN25 WD 11). As the objective is specifically built for dark-field illumination, it has a separate light path for the incident light, at the end of which a ring-shaped lens focuses the light in the focus spot of the imaging path. Here, a set of lenses collects the scattered light into a collimated beam that then is directed into the spectrometer (Andor SR-193I-A-SL), where a grating (150 l/mm) separates the individual wavelength components. The spectra are finally recorded by a CCD camera (Andor Newton DU920P-BEX2-DD).*

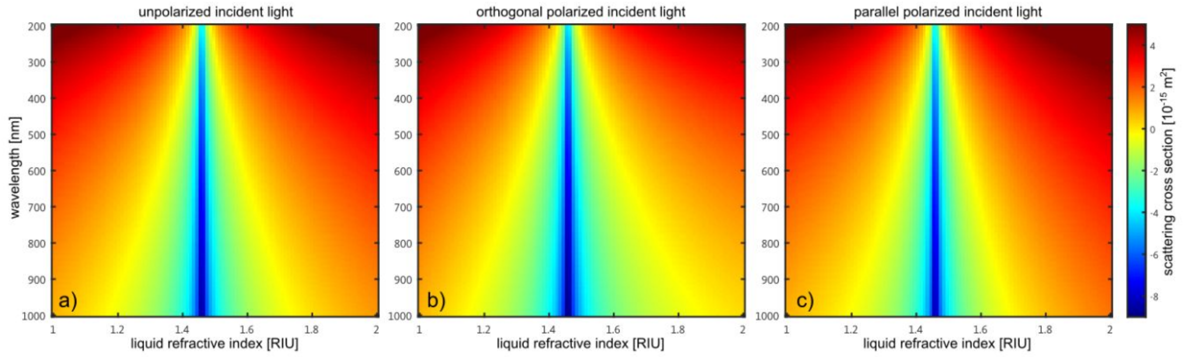

Figure S2. ***Nanochannel scattering cross section dependence on wavelength and RI.*** *The three panels show how the scattering cross section, of a 200 nm by 200 nm nanochannel embedded in SiO_2 (for which the RI is calculated for each wavelength according to Malitson et. al.[1]) depends on the wavelength of the incident light and the RI of the medium that fills the channel, for different polarizations of the incident light respectively. The color code axis is given in logarithmic units with base ten. a) For unpolarized light, the scattering cross section of the channel ($\sigma_{channel}$) shows a minimum around the RI of SiO_2, i.e. n = 1.459 at 600 nm, since in this case the channel and its surrounding medium form an optically uniform body. Deviations from this value lead to an increase in the $\sigma_{channel}$ for all wavelengths. For shorter wavelengths, $\sigma_{channel}$ increases more rapidly to higher values. For RIs of the channel that are larger than the surrounding material, $\sigma_{channel}$ increases slightly more than when compared to the same RI difference for a channel with lower RI (see also* ***Figure 1e*** *in the main text). b) For light that is polarized orthogonal to the channel, the overall result is the same as in a), with the exception that $\sigma_{channel}$ is not as large as in the unpolarized case. c) For light that is polarized parallel to the nanochannel the overall $\sigma_{channel}$ is larger than in the other two cases. Especially remarkable here is the increase of $\sigma_{channel}$ when the channel has a higher RI than the surrounding material, which becomes even more significant when considering shorter wavelengths.*

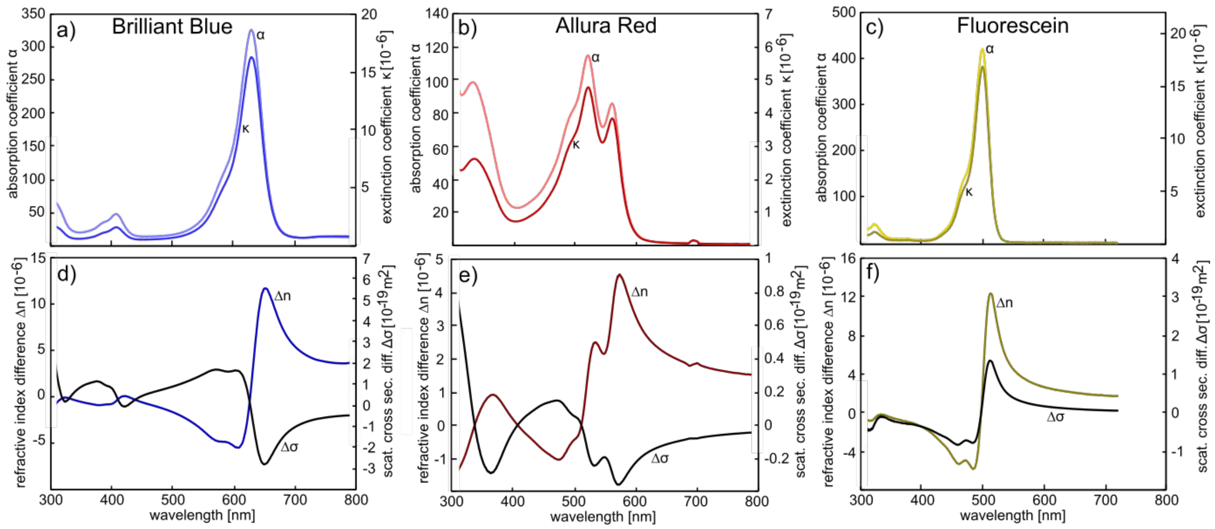

Figure S3. ***Spectra of Brilliant Blue, Allura Red and Fluorescein during the analytical transformation from absorption spectra to scattering spectra.*** *a) to c) The absorbance spectra as measured with absorption spectrophotometry (ASP) using an Varian Cary 50 Bio instrument are here shown as absorption coefficient spectra, which can be calculated by dividing the measured absorbance values by the optical path through the sample solution (here 1 cm) and by log(e). The subsequent calculation of the extinction coefficient κ is done with* ***Equation 3*** *in the main text. d) to f) Using κ in the Kramers-Kronig relation (****Equation 4*** *in the main text) yields the change of the real part of the refractive index (RI) caused by the respective absorption features of each type of dye molecule in the solution. Applying then* ***Equation 1*** *from the main text delivers the scattering cross section, $\sigma_{channel,solution}$, of a nanochannel filled with the corresponding dye solution and corresponding concentration. Subtracting from $\sigma_{channel,solution}$ the scattering cross section, $\sigma_{channel,water}$, of a water-filled channel yields the difference in scattering cross section, Δσ, which is proportional to the observed difference in scattering intensity (RSID). The calculated spectra for Δσ are used as a qualitative benchmark in the comparison with the experimental RSID spectra since the dye concentrations used in the nanochannels are too high to be measured with ASP.*

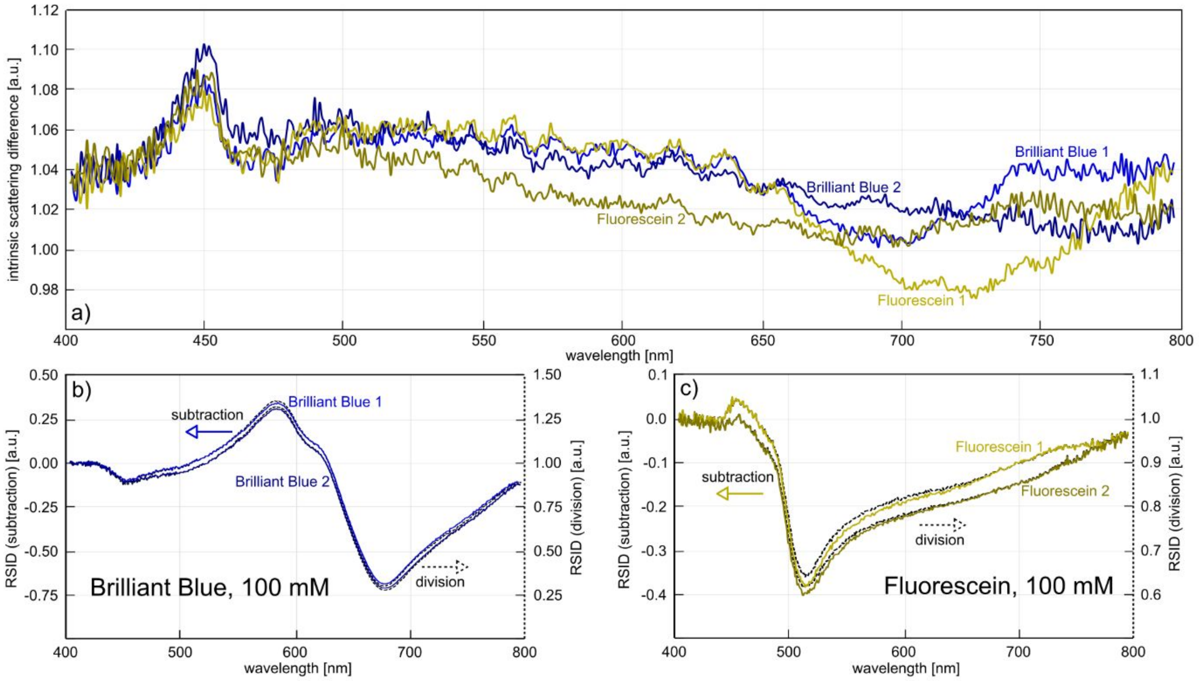

Figure S4. ***Intrinsic scattering difference and comparison of evaluation strategies.*** *a) Intrinsic scattering difference spectra for two independent measurement series of the dyes Brilliant Blue and Fluorescein (100 mM) as used for* ***Figure 6b,d*** *and* ***Figure 8b****. The intrinsic scattering difference is here defined as the ratio of the scattering intensity of a water-filled sample channel and the scattering intensity of its corresponding water-filled reference channel. The colors indicate to which of the two subsequent RSID measurements of Brilliant Blue (blue) and Fluorescein (yellow) they belong, which are shown in b) and c) respectively. b) RSID spectra for two independent measurements of a 100 mM Brilliant Blue solution in a 200 nm by 200 nm channel. The solid lines show the result when the intrinsic scattering difference is subtracted (as described in the main text,* ***Figure 4****), while the dashed lines are the result when instead division by the intrinsic scattering difference is used. c) Same as for b), however, for a 100 mM Fluorescein solution.*

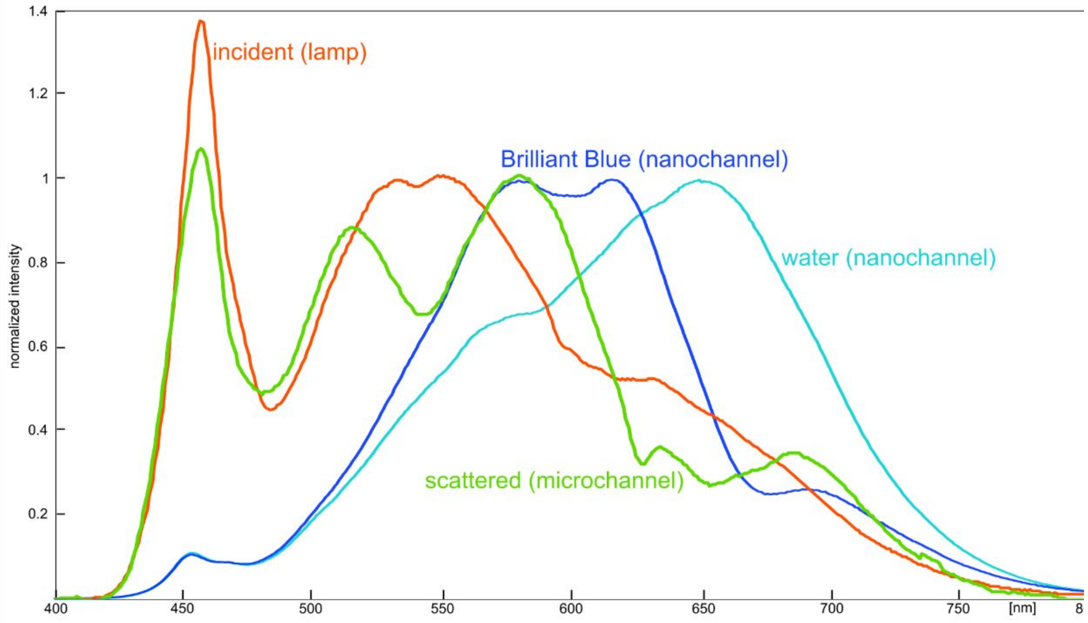

Figure S5. ***Spectra of the light used during the experiment, scaled to their maximum between 500 nm and 700 nm.*** *The initial incident light spectrum (red line) measured with an Avantes AvaSpec-1024 spectrometer is emitted from a Thorlabs Solis-3C LED light source. It undergoes several changes regarding the shape of the spectrum during its course through the experimental setup. The light source is a broad-band LED-lamp, that has its main intensity centered around 550 nm but also an additional strong peak at 450 nm. After being focused on the fluidic system, the light scatters on the fluidic channels (green line, scattering from a microchannel wall for intensity reasons, recorded with an Avantes AvaSpec-1024 spectrometer). Here we see that the spectral shape of the lamp is not maintained. Most remarkable is the dip that has appeared at 540 nm but also the peak at 450 nm has decreased in relative size. We assume that thin-film interference in the* SiO_2 *layer of the fluidic chip causes these changes. After being scattered from a water-filled nanochannel (cyan line, measured with a Andor SR-193I-A-SL spectrometer and a Andor Newton DU920p-BEX2-DD camera), the shape of the spectrum has again changed, now being more bell-shaped with a maximum at 650 nm. We see the main cause for this in the sensitivity of the grating spectrometer and the camera. The peak at 450 nm is now greatly diminished when compared to the original spectrum. When the nanochannels are now filled with a dye (Brilliant Blue as example), the spectrum changes again in shape according to the change in absorption and RI as explained in the main text. All spectra have been normalized to their maximum value in the wavelength range over 500 nm.*

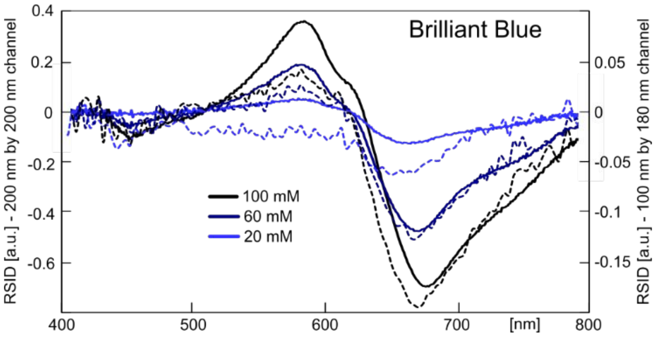

Figure S6. ***Comparison of RSID spectra measured from nanochannels with different dimensions.*** *RSID spectra for three different Brilliant Blue concentrations measured in a nanochannel with 200 nm x 200 nm cross section (continuous lines) a nanochannel with 100 nm x 180 nm cross section (dashed lines).*

Section II: Supplementary derivations

Calculation of the molar extinction coefficient from measured RSID spectra

In the main text, we explained how the expected relative scattering intensity difference (RSID) spectrum can be calculated from the absorbance spectra of the respective dyes. Here, we will outline the reverse process leading to **Figure 9** in the main text. As a first step, we need to establish the connection between RSID and scattering cross section of the nanochannel, $\sigma_{channel}$. The scattered power, P_{scat}, is the product of incident intensity, $I_{incident}$, and $\sigma_{channel}$. The RSID as shown in the main text is the scattering spectra recorded from the solution-filled channels divided by the scattering spectra from the water-filled channel but then also with the *intrinsic difference spectrum* subtracted. To amend this, we add an ideal *intrinsic difference spectrum* for a ratio, which is 1, to the RSID spectra (see **Figure S7a-c**). Here, the shaded area below 425 nm for all graphs indicates the wavelength range where the intensity of the incident light and the sensitivity of the spectrometer and camera is insufficient to conduct a measurement of RSID.

$$RSID + 1 = \frac{P_{scat,\,solution}}{P_{scat,\,water}} = \frac{I_{incident}\sigma_{channel,solution}}{I_{incident}\sigma_{channel,water}} = \frac{\sigma_{channel,solution}}{\sigma_{channel,water}} \qquad \textit{Equation S1}$$

As second step, we consider the scattering cross section, $\sigma_{channel}$, of a nanochannel as given by **Equation 1** in the main text, but as a simplification consider here only incident light that is polarized parallel to the nanochannel, as it is the main contributor to the scattering intensity (see **Figure 1e**).

$$\sigma_p = \frac{A_\emptyset^2 k^3 L}{4}(m^2 - 1)^2. \qquad \textit{Equation S2}$$

With this expression at hand, we can write the RSID as a ratio of scattering cross sections, were the geometry and wavelength dependent pre-factor vanishes, and were we substitute m again as the ratio of RIs of the solution in the channel and the SiO_2 the channel is embedded in.

$$RSID + 1 = \frac{\sigma_{channel,p,\,solution}}{\sigma_{channel,p,water}} = \frac{\left(\left(\frac{n_{solution}}{n_{SiO2}}\right)^2 - 1\right)^2}{\left(\left(\frac{n_{water}}{n_{SiO2}}\right)^2 - 1\right)^2} \qquad \textit{Equation S3}$$

Solving this equation for $n_{solution}$ provides four solutions, of which we will continue with the following as it is not resulting in negative values for $n_{solution}$and applies to the case where $n_{water} < n_{SiO2}$.

$$n_{solution} = \sqrt{\sqrt{(RSID + 1)\left(n_{water}^2 - n_{SiO2}^2\right)^2} + n_{SiO2}^2} \qquad \textit{Equation S4}$$

Using here the literature values for the RIs for water[2] and SiO_2[1], we arrive at the RI spectra for the respective solutions, as shown in **Figure S7d-f**. It is seen clearly that the dye solutions have RIs larger than water ($n_{H2O} = 1.333$ at 600 nm). The absorption bands of the dyes appear as deviations from the smooth, Cauchy-type curve (yellow dashed line) fitted to the RI spectra. In the presented calculation, it is assumed that the respective dye solutions do not have any absorption in this regime and follow a normal Cauchy dispersion. This assumption enables the fitting of a Cauchy-type function in **Figure S7d-f**. This fit is necessary for the next step of our transformation of RSID to molar extinction coefficient, since only the RI features caused by the absorption bands of the dyes are of interest in the Kramers-Kronig relation (and the corresponding inverse relation) and since the RI spectrum, in addition to the features related to the absorption bands, contains contributions that are not related to absorption, which we need to subtract that part using the Cauchy-fit to estimate it.

The Cauchy-formula is commonly used as a simple description of the normal dispersion relation of transparent media in the visible regime, making it useful to us here to separate normal from anomalous dispersion (which is the extinction of the dye). To do so, we need to note that due to the fact that the Kramers-Kronig relation contains an integral, the whole RI spectrum experiences a change because of the anomalous dispersion, which is most pronounced for the longer wavelengths[3]. The Cauchy-fit is therefore based on the section of the RI curve that is below 400 nm. Furthermore, to include the overall increase of the RI for all wavelengths as mentioned above, we estimated this contribution to be 0.01 RI units and subtracted it from the initial fit (grey dashed line in **Figure S7d-f**) such that the extracted contribution of the normal dispersion (grey line) happens to be lower than the baseline of the calculated RI spectrum of the dyes. This estimate seems to be reasonable, as it is in line with our understanding of the Kramers-Kronig relation for the normal and anomalous dispersion of a dye solution and reproduces the expected result sufficiently well.

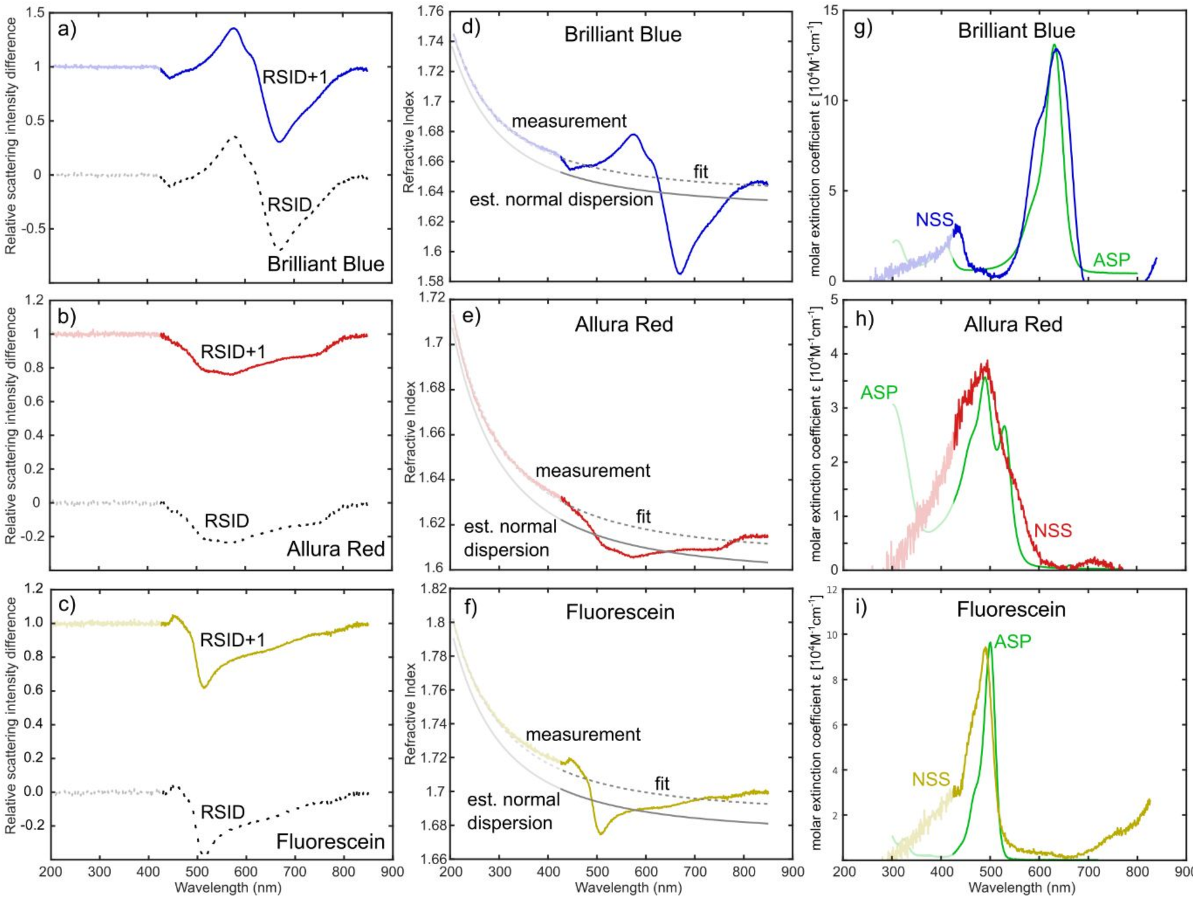

Figure S7. ***Translation of RSID to molar extinction coefficient.*** *The shaded sections of the spectra indicate the wavelength regime where low incident light intensity and insufficient spectrometer and camera sensitivity prevent a measurement of RSID. a)-c) The RSID spectra from the experiment (**cf. Figure 6b-d**, dashed lines) for a 100 mM Brilliant Blue, Allura Red and Fluorescein solution, respectively. The colored solid lines are RSID+1 spectra and represent the ratio of scattering cross sections as laid out in **Equation S1**. d)-f) Using **Equation S4**, the RSID (which is the ratio of scattering cross sections) can be translated into the RI spectrum of the respective dye solution (colored lines). To extract the part of the RI spectrum that describes normal dispersion and that is not associated with the absorption bands of the solute, a Cauchy-type fit (grey dashed line) is applied to the RI spectrum and the estimated contribution of the normal dispersion (grey line) is later subtracted from the RI curve of the solution. g)-i) Molar extinction coefficient spectra for the three dyes obtained by reverse Kramers-Kronig transformation (colored lines, **Equation S5**) plotted together with the molar extinction coefficient spectra obtained for the same dyes using traditional ASP (green lines). We note the generally good agreement between NSS-based and ASP spectra. The unphysical negative molar extinction coefficients obtained by converting the Brilliant Blue NSS data (g) between 700 nm and 800 nm, comes*

most probably from the long-wavelength contribution of the anomalous dispersion to the normal dispersion that is not represented in the Cauchy-fit The molar extinction coefficients also become negative for all dyes below 300 nm, but here we assume that the high noise and low intensity of the scattered signal is responsible for this unphysical deviation.

The inverse Kramers-Kronig relation for the real and imaginary part of the RI (of which the imaginary part correspond to extinction) is nearly identical in structure to **Equation 4** in the main text, except for a minus sign in front and can be used to calculate the wavelength-dependent extinction coefficient as

$$\kappa(\lambda) = -\frac{2}{\pi}\mathcal{P}\int_{0}^{-\infty} \frac{\Delta n(\lambda')}{\lambda'\left(1-\left(\frac{\lambda'}{\lambda}\right)^{2}\right)} d\lambda' \qquad \textit{Equation S5}$$

As the very last step, we can then convert the obtained extinction coefficient spectrum , $\kappa(\lambda)$, into the molar extinction coefficient spectrum, $\varepsilon(\lambda)$ by using the known concentration of $c = 100\, mM$ in **Equation S6** for each solution.

$$\varepsilon(\lambda) = \frac{4\pi\kappa(\lambda)}{\lambda\, c} \qquad \textit{Equation S6}$$

The final result of this calculation is shown in **Figure S7g-i**, together with the molar extinction coefficients for the respective dyes that have been measured using ASP. The agreement is relatively good regarding the main peak positions and the maximum value of the molar extinction coefficient ε for all three dyes. The values for ε do deviate from the ASP spectra slightly but we identify the main reason for this in the separation of the normal and anomalous dispersion as explained above. The Cauchy-fit of the RI spectrum for each respective dye solution may not represent the actual underlying normal dispersion (associated with the real part of the RI of the solution), as it may not fully represent all involved influences of the dye extinction on the total RI. For the shorter wavelengths below 300 nm, poor signal quality due to low scattering intensity can also be considered as reason for these deviations. Furthermore, the RI of the surrounding SiO_2 plays a critical role during the calculation, since even a small change in the chosen value has a sizable impact on the final result, since NSS is as sensitive to the RI in the channel as to the RI outside of the channel, as laid out by **Equation 1** in the main text.

References

1. Malitson, I. H. Interspecimen Comparison of the Refractive Index of Fused Silica. *J. Opt. Soc. Am.* **55**, 1205–1209 (1965).

2. Hale, G. M. & Querry, M. R. Optical Constants of Water in the 200-nm to 200-μm Wavelength Region. *Appl. Opt.* **12**, 555–563 (1973).

3. Sai, T., Saba, M., Dufresne, E. R., Steiner, U. & Wilts, B. D. Designing refractive index fluids using the Kramers-Kronig relations. *Faraday Discuss.* **223**, 136–144 (2020).
